# Loading is not delivering: Intracellular drug retention and migration capacity govern macrophage-based drug delivery

**DOI:** 10.1126/sciadv.aec0429

**Published:** 2026-07-24

**Authors:** Yao Huang, Jinhui Lin, Yuhang Long, Shuai Zheng, Xiangliang Yang, Jouni Hirvonen, Yaping Ding, Wei Li

**Affiliations:** ^1^ National Engineering Research Center for Nanomedicine, College of Life Science and Technology, Huazhong University of Science and Technology, Wuhan 430074, China.; ^2^ Drug Research Program, Division of Pharmaceutical Chemistry and Technology, Faculty of Pharmacy, University of Helsinki, Helsinki 00014, Finland.; ^3^ Hubei Key Laboratory of Bioinorganic Chemistry and Materia Medica, Huazhong University of Science and Technology, Wuhan 430074, China.

## Abstract

Living cells are increasingly explored as drug carriers for their active targeting capacity, yet robust intracellular loading in vitro often fails to translate into efficacy in vivo. Here, we address this disconnect by loading macrophages with nano- and microdrugs with equal intracellular mass and matched release kinetics. Quantitative tracking revealed that in vitro retention overestimates in vivo stability: Nanodrug-loaded macrophages lost up to 83% of their payload after reinfusion, nearly double the loss observed in vitro. Nanodrug loading also elevated oxidative stress in macrophages, thereby impairing chemotactic migration. By contrast, microdrugs persisted intracellularly for ≥7 days and preserved macrophage migration toward inflamed sites. Using intravital imaging, we provided previously unavailable spatiotemporal evidence of vascular transmigration while retaining cargo. These properties translated into superior efficacy in systemic and localized inflammation models. Our findings underscore that effective cell-based drug delivery requires looking beyond uptake to evaluate in vivo cargo persistence and its impact on cell migration.

## Teaser

Looking beyond uptake, microdrugs keep cells loaded and moving to targets.

## INTRODUCTION

Cell-based drug delivery systems (CDDSs) promise to harness the homing ability and functional versatility of living cells to improve drug targeting and amplify the therapeutic functions of cells (*1*, *2*). Advances in pharmaceutical formulation have markedly increased intracellular drug loading, often surpassing free drug molecules by orders of magnitude (*3*). Once internalized, the endogenous biochemical environment of carrier cells, such as enzyme activity, pH, and redox conditions, can serve as intracellular triggers for spatiotemporal drug release (*4*). The released drug may, in turn, modulate cell behavior (e.g., migration and phenotype), creating a feedback loop between the drug and the carrier cells. This mutual reinforcement offers a unique pharmacological advantage over conventional drug formulations. Yet, despite these advantages, a critical limitation persists: What is efficiently loaded in vitro is often not what is faithfully delivered in vivo. Premature cargo expulsion and impaired cell migration together disrupt the linkage between loading and delivery, undermining the translational fidelity of CDDS and representing a field-wide barrier that has yet to be resolved.

Retention of intracellular payloads represents the first prerequisite for delivery fidelity. Nanodrugs, such as nanoparticles, have been widely loaded into cells due to their high uptake efficiency. However, high uptake does not guarantee successful delivery. Multiple in vitro studies have shown that nanodrugs are rapidly exocytosed through lysosomal-based (*5*) or endoplasmic-Golgi apparatus-based pathways (*6*), commonly resulting in premature loss and off-target distribution. Microdrugs, by contrast, display prolonged intracellular retention in preclinical in vitro studies (*7*–*9*), and strategies such as intracellular self-assembly of nanostructures into larger aggregates (*10*) or direct microparticle loading have been explored to exploit this advantage (*9*). Nevertheless, the extent to which intracellular payloads, be they nanodrugs or microdrugs, can be stably retained and faithfully delivered in vivo remains poorly understood, as carrier cells are exposed to shear stress, biochemical stimuli, and systemic dilution that often exceed in vitro levels.

Chemotaxis constitutes the second requirement for effective CDDS, as the homing of circulating immune cells dictates whether retained intracellular drugs can be delivered to pathological sites. Nanodrugs are generally assumed to exert minimal impact on cell motility. Supporting this notion, studies have reported that various nanoparticles, such as silica nanocapsules in macrophages (*11*) and liposomes in neutrophils (*12*), do not notably alter the cell migration behavior compared to unloaded controls. Certain nanoparticles may even appear to enhance cell migration under external cues (*13*). However, other studies have shown that TiO_2_ (*14*) and gold nanoparticles (*15*) reduce macrophage migration speed by over 50%, largely due to oxidative stress, nuclear stiffening, and cytoskeletal dysregulation (*14*–*16*). Conversely, microdrugs (1 to 3 μm), once considered suboptimal to cross the vascular barrier, have shown unexpectedly superior ability to traverse the blood-brain barrier owing to enhanced interfacial interactions and endocytic uptake (*17*). Intriguingly, certain live micrometer-sized cargos, such as bacteria, can even promote host cell migration by enhancing their viability under mechanical stress (*18*). These contradictory findings hinder our understanding of whether CDDSs preserve the native homing capabilities of their carrier cells or exhibit altered trafficking dynamics, a key determinant of their ultimate therapeutic efficacy.

**Fig. 1.**
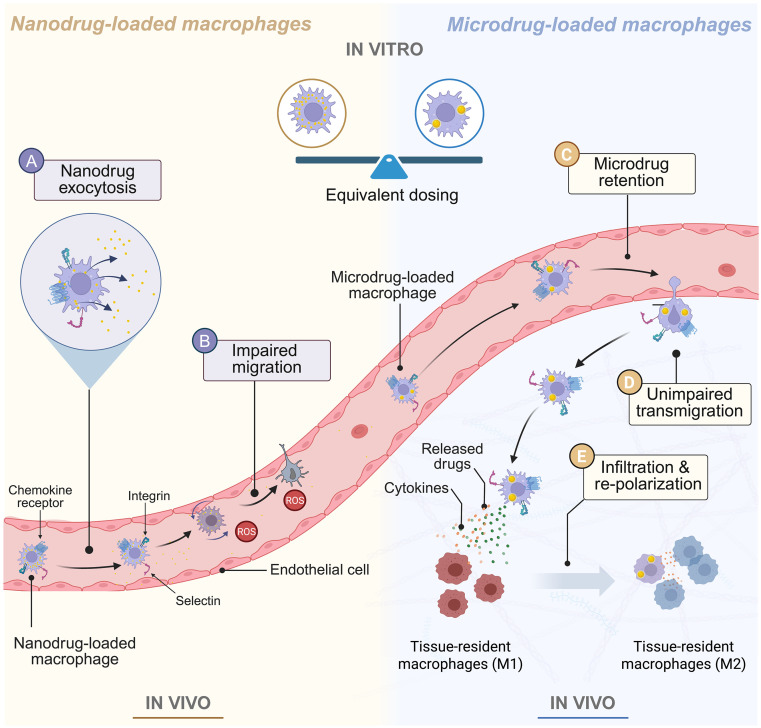
Schematic illustration of the translation from in vitro drug loading to in vivo drug delivery by macrophages. Following intracellular loading in vitro, nanodrugs undergo rapid exocytosis (**A**) and induce intracellular accumulation of reactive oxygen species (ROS), thereby impairing chemotactic migration (**B**) in vivo. In contrast, microdrugs exhibit prolonged intracellular retention (**C**), preserve the native migratory capacity of carrier macrophages (**D**), and enable effective infiltration into inflamed tissues, thereby supporting the repolarization of both carrier and resident macrophages (**E**). Created in BioRender. Huang, Y. (2026) https://BioRender.com/h3a9u8x.

In this study, we systematically dissect what determines the disconnect between in vitro loading and in vivo delivery, and how intracellular payload design regulates retention, carrier cell migration, and therapeutic efficacy. To this end, we developed monodisperse nano- and microparticles as two representative classes of payloads, including drug-free poly(lactic-*co*-glycolic acid) (PLGA) particles for mechanistic studies and dexamethasone palmitate (DexP) particles for therapeutic evaluation. Formulations were adjusted to achieve comparable intracellular loading, thereby generating nanodrug- and microdrug-loaded macrophages for direct comparison. This controlled design enabled us to trace the intracellular fate of different payloads, quantify their persistence across in vitro and in vivo contexts, and assess their impact on macrophage chemotaxis. By integrating flow cytometry, intravital imaging, transcriptomic profiling, and disease models, we revealed how nanodrugs and microdrugs differentially govern intracellular retention, migration dynamics, and therapeutic efficacy. Through this framework, we provide the quantitative and mechanistic evidence that intracellular payload form dictates whether carrier cells can translate loading into faithful delivery. By uncovering retention and migration as the dual prerequisites for fidelity, we redefine the design principles of CDDS and establish microdrug loading as a “load once, deliver fully” strategy. (Fig. 1)

## RESULTS

### Intracellular drug loading in vitro does not guarantee stable retention in vivo

To test whether intracellular drug loading in vitro reliably predicts retention in vivo, we used microfluidics to generate monodisperse PLGA particles with precisely controlled diameters of 0.2, 1, and 3 μm. These sizes were strategically selected to represent the nano- to micro-sized spectrum: 0.2 μm as a common nanocarrier, 1 μm as a transitional intermediate, and 3 μm as the upper limit typically internalized by macrophages through phagocytosis.

**Fig. 2.**
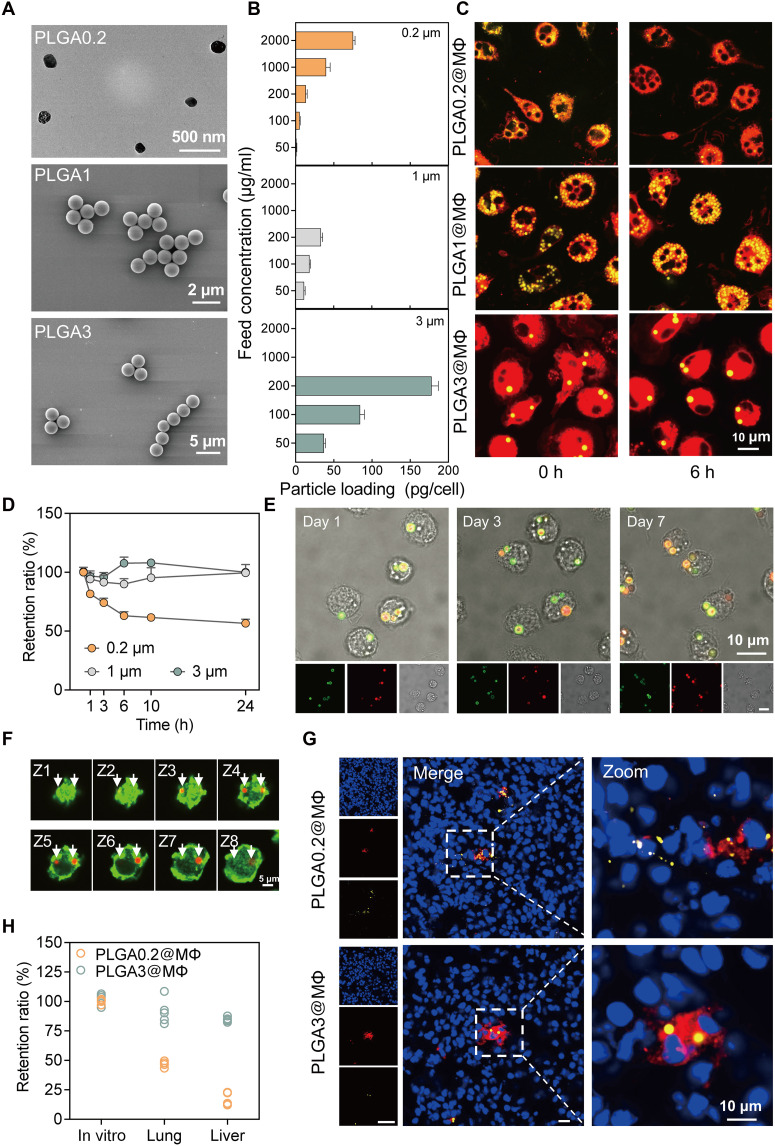
In vitro retention overestimates in vivo stability. (**A**) Morphology of PLGA particles: (i) 0.2 μm [PLGA0.2, transmission electron microscopy (TEM), scale bar: 500 nm], (ii) 1 μm (PLGA1, scanning electron microscopy (SEM), scale bar: 2 μm), and (iii) 3 μm (PLGA3, SEM, scale bar: 5 μm). (**B**) Intracellular loading of 0.2-, 1-, and 3-μm PLGA particles after 4-hour (h) incubation (*n* = 3). (**C**) Intracellular retention of PLGA particles in macrophages observed by confocal laser scanning microscopy (CLSM) at 0 and 6 hours (red: cell membrane; yellow: PLGA-RhB particles; scale bar: 10 μm). (**D**) Exocytosis kinetics of 0.2-, 1-, and 3-μm PLGA particles in macrophages quantified by flow cytometry (*n* = 3). (**E**) Intracellular retention of 3-μm PLGA particles under in vitro conditions. (CLSM, bright-field: macrophage; green: lysosome; red: PLGA-RhB particles, microparticles are pseudocolored in red to improve visual contrast; scale bar: 10 μm). (**F**) Sequential *Z*-slices showing the actin cytoskeleton of macrophages loaded with 3-μm microparticles after the 4-hour loading period (F-actin is pseudocolored in green and microparticles in red to improve visual contrast, scale bar: 5 μm). (**G**) Lung cryosection collected at 24 hours after adoptive transfer of PLGA0.2@MΦ and PLGA3@MΦ (CLSM, red: cell membrane; yellow: PLGA-RhB particles; blue: nucleus; scale bar: 50 μm, Scale bars in merged and zoomed images: 10 μm). (**H**) Intracellular retention of 0.2- and 3-μm PLGA particles measured in vitro before adoptive transfer and in macrophages that accumulate at the lung and liver 24 hours after adoptive transfer (flow cytometry, *n* = 5).

All particles were spherical and highly uniform, as confirmed by electron microscopies (Fig. 2A). The 0.2-μm nanoparticles showed a narrow hydrodynamic size distribution [200.0 ± 7.5 nm, polydispersity index (PDI) = 0.09 ± 0.01] (fig. S1), while the 1- and 3-μm microparticles had mean diameters of 1.03 ± 0.04 μm and 3.01 ± 0.10 μm, respectively, with coefficients of variation below 4%, indicating excellent size control.

We first evaluated uptake by incubating bone marrow-derived macrophages (BMDMs, MΦ) with each particle type. Intracellular particle mass was quantified by correlating fluorescence intensity of cell lysates with a standard calibration curve (fig. S2). Despite comparable biocompatibility (fig. S3), microparticles achieved considerably higher intracellular payloads than nanoparticles at the same dosing concentration (Fig. 2B). This difference stems from the markedly greater mass per microparticle: A single 3-μm particle equates to the mass of 27 particles at 1 μm or 3375 particles at 0.2 μm. Consequently, fewer microparticles are required to achieve a given mass uptake, thereby imposing a lower burden on endocytic processing. To eliminate uptake bias and focus on retention, we adjusted the dosing conditions to ensure that macrophages internalized an equivalent particle mass (∼35 pg per cell) across all groups.

Under equivalent intracellular payload, retention kinetics exhibited a strong size dependence. Nanoparticles were rapidly exocytosed, with 43.4% eliminated within 10 hours (Fig. 2, C and D), primarily via lysosome-mediated exocytosis pathways (fig. S4). In contrast, microparticles remained stably retained within phagolysosomes for at least 7 days with negligible efflux (Fig. 2, D and E). Notably, there were no signs of lysosomal escape (Fig. 2E) or actin polymerization–based transport typically exploited by pathogens (Fig. 2F), indicating robust intracellular localization and stability. These in vitro findings offer important insights into cargo-dependent retention.

However, such models do not fully replicate the complex physiological environment of living systems, a critical limitation in translational drug delivery research. On the basis of our in vitro screening, we selected 0.2- and 3-μm particles for subsequent in vivo evaluation as they represent two functionally distinct regimes, whereas 1-μm microparticles were excluded due to functional overlap with the 3 μm group. We adoptively transferred DiD-labeled BMDMs loaded with either 0.2- or 3-μm particles [2 × 10^6^ cells in 100 μl of phosphate-buffered saline (PBS)] into mice with systemic inflammation within 30 min of cell collection (fig. S5). Owing to the high cell density and low temperature (4°C) during transit, no appreciable intracellular nanoparticle loss was detected before reinfusion (fig. S6). Ex vivo imaging via in vivo imaging system (IVIS) confirmed predominant macrophage accumulation in the liver and lung (fig. S7, A and B). At day 1 postadoptive transfer, PLGA0.2@MΦ were predominantly distributed in the liver (68%), with 21% detected in the lung. By day 5, the liver-associated fraction of PLGA0.2@MΦ remained relatively stable (∼65%), whereas the lung fraction decreased to 14.5%, accompanied by increased redistribution to other organs (heart, spleen, and kidney) (fig. S7, A and C). In contrast, PLGA3@MΦ exhibited a lower liver fraction (54%) and a higher lung fraction (37%) at day 1, consistent with transient pulmonary retention of macrophages carrying larger intracellular particles, which require more time to traverse the narrow pulmonary capillary network (*19*). By day 5, PLGA3@MΦ redistributed systemically, with the liver fraction increasing to 64% and the lung fraction decreasing to 14.2% (fig. S7, B and D), similar to PLGA0.2@MΦ.

Particle cargo displayed size-dependent biodistribution dynamics. PLGA0.2 nanoparticle localization progressively diverged from cellular distribution: While nanoparticle signal largely matched PLGA0.2@MΦ localization at day 1 (liver 66.5% and lung 21%), by day 5, the liver-associated fraction decreased to 48% and the lung-associated fraction increased to ∼30%, markedly exceeding the corresponding lung fraction of PLGA0.2@MΦ cells (∼14.5%) (fig. S7, A and C). This may be due to the longer time required for macrophages to traverse the pulmonary capillary network and cell redistribution between organs, which increases the accumulation of exocytosed nanoparticles in the lung and decreases that in the liver, respectively. These data indicated time-dependent spatial decoupling of nanoparticle cargo from carrier macrophages, particularly within the pulmonary compartment. In other organs, nanoparticle fluorescence followed trends similar to those of the carrier macrophages, suggesting that the majority of released nanoparticles remained within tissues accessed by the carrier cells. In contrast, PLGA3 microparticle fluorescence closely mirrored the distribution of carrier macrophages at both time points, indicating sustained intracellular retention (fig. S7, B and D).

Consistently, tissue cryosections revealed that nanoparticles were predominantly localized extracellularly, indicative of intracellular cargo loss in vivo, whereas microparticles remained stably confined within carrier macrophages (Fig. 2G). Flow cytometry analysis of digested organs corroborated these findings: 53.8 ± 2.7% of nanoparticles were expelled by particle-loaded adoptively transferred macrophages that accumulated in the lungs, increasing to 83.2 ± 5.4% in the liver (see the gating method in figs. S8 and S9), representing almost 1.2- to 2.0-fold higher efflux compared to in vitro conditions. In contrast, over 85% of microparticles remained intracellular within carrier macrophages in both the lung and the liver (Fig. 2H). Notably, given the early time window analyzed, the observed signal loss is unlikely to be driven by PLGA degradation, which typically occurs over days to weeks (*20*, *21*), and instead primarily reflects efflux and subsequent clearance in vivo.

Together, these results establish that intracellular drug loading in vitro does not guarantee stable retention in vivo. Instead, the physiological environment amplifies exocytosis in a payload-dependent manner, accelerating loss of nanoparticles while sparing microparticles. This discrepancy highlights that evaluating CDDS solely under static culture conditions in vitro can be misleading, and that drug properties must be considered in the context of in vivo physiological stressors to ensure delivery fidelity.

### Stable intracellular drug persistence ensures accurate dosing and sustained macrophage anti-inflammatory function

**Fig. 3.**
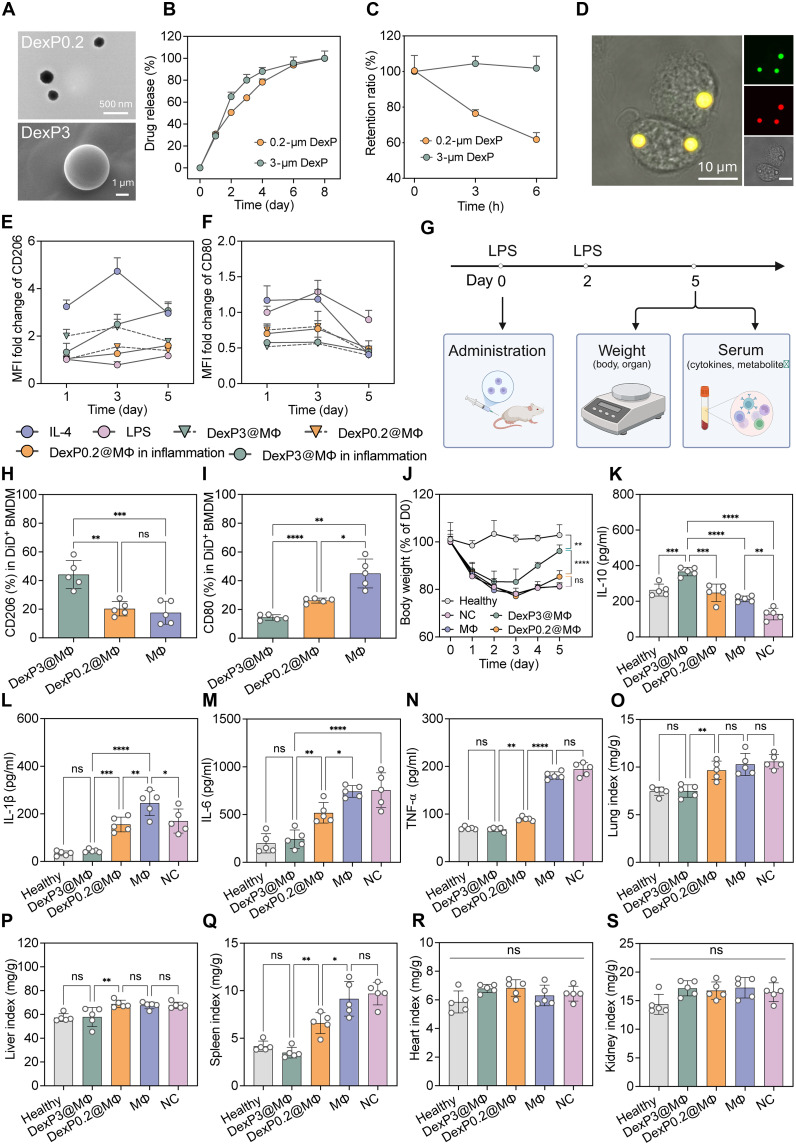
Intracellular anti-inflammatory microparticles enable persistent M2 macrophage polarization and systemic inflammation amelioration. (**A**) Morphology of DexP particles: (i) 0.2 μm (DexP0.2, TEM, scale bar: 500 nm), (ii) 3 μm (DexP3, SEM, scale bar: 1 μm). (**B**) Drug release of 0.2- and 3-μm DexP particles in response to lipase (*n* = 3). (**C**) Exocytosis kinetics of 0.2- and 3-μm DexP particles in macrophages quantified by flow cytometry (*n* = 3). (**D**) Intracellular retention and lysosomal colocalization of DexP particles at day 3 observed by CLSM (bright-field: macrophage; green: lysosome; red: DexP-DiI microparticles; scale bar: 10 μm). (**E** and **F**) CD206 (M2 marker) and CD80 (M1 marker) expression in macrophages (MΦ, DexP0.2@MΦ, and DexP3@MΦ) quantified by flow cytometry (*n* = 3). (**G**) Schematic illustration of systemic inflammation therapy. Created in BioRender. Huang, Y. (2026) https://BioRender.com/srau444. (**H** and **I**) Flow cytometry quantification of CD206 and CD80 expression in adoptively transferred macrophages (MΦ, DexP0.2@MΦ, and DexP3@MΦ) recovered from liver tissue (*n* = 5). (**J**) Body weight of mice after specified treatment (*n* = 5). (**K** to **N**) Serum levels of anti-inflammatory and proinflammatory cytokines (*n* = 5). (**O** to **S**) Organ index of major organs (*n* = 5). *P < 0.05, **P < 0.01, ***P < 0.001, and ****P < 0.0001; data are shown as mean ± SD, statistical significance was analyzed using a one-way ANOVA with Tukey’s multiple comparisons [(H), (J) to (S)], or Brown-Forsythe and Welch ANOVA tests, followed by Dunnett’s T3 multiple comparisons due to unequal variances (I).

The prolonged intracellular retention of microparticles enables sustained maintenance of the intracellular dose throughout the delivery period. To evaluate whether this intracellular dose accuracy translates into therapeutic benefits, we developed a delivery platform in which nanoparticles and microparticles shared identical drug composition, loading content, and release kinetics. Enzyme-responsive prodrug (DexP) was used to enable spontaneous self-assembly into uniform particles of defined sizes (Fig. 3A and fig. S10). Under lipase stimulation, both formulations exhibited comparable near-zero-order release over 1 week (Fig. 3B). This is likely due to the porous internal structure of the microparticles, which permitted enzyme penetration and enhanced the enzyme-responsive release kinetics, thereby rendering the release rate comparable to that of nanoparticles. Similarly, to isolate the effect of retention kinetics, intracellular DexP levels were equalized to 20.7 ± 2.5 pg/cell across groups (fig. S11). Consistent with earlier findings, DexP nanoparticles were rapidly cleared via exocytosis, whereas microparticles demonstrated prolonged intracellular retention (Fig. 3, C and D). Notably, lysosomal tracking revealed that the probe fully infiltrated DexP microparticles rather than remaining confined to the particle periphery, further supporting the presence of an internal porous structure that facilitates enzyme access (Fig. 3D). These results confirm that intracellular retention is governed primarily by drug size, independent of material composition.

To assess the functional implications of accurate intracellular dose, we analyzed the polarization of carrier macrophages. In the absence of external stimulation, microparticle-loaded macrophages exhibited sustained up-regulation of the M2 marker CD206, peaking at 2.4-fold on day 3 and remaining elevated (1.8-fold) on day 5, consistently outperforming the nanoparticle group, which peaked at 1.6-fold on day 3. Under lipopolysaccharide (LPS) challenge, this disparity became more pronounced: microparticles induced a continuous increase in CD206 expression, reaching 3.1-fold by day 5, nearly double that of the nanoparticle group. By comparison, interleukin-4 (IL-4)–treated controls showed a sharp drop (>50%) in CD206 expression between day 3 and day 5, reflecting cytokine depletion and highlighting the advantage of intracellular drug retention (Fig. 3E). CD80, an M1 marker, was concurrently down-regulated in the microparticle group (Fig. 3F). Cytokine profiling supported these trends, revealing elevated IL-10 and reduced levels of IL-6, tumor necrosis factor–α (TNF-α), and IL-1β secreted by microparticle-loaded macrophages (fig. S12).

To assess whether enhanced polarization translates into therapeutic efficacy, we used a murine model of systemic inflammation (Fig. 3G). This model establishes a uniform inflammatory context that yields comparable macrophage distribution across major organs (fig. S7), thereby reducing confounding variability. An equal number of macrophages containing equivalent intracellular DexP doses were adoptively transferred across groups. This design ensured the same systemic drug exposure, isolating intracellular drug retention as the only variable and enabling a focused evaluation of its impact on therapeutic outcomes.

To capture the early phenotypic state of adoptively transferred macrophages under inflammatory conditions in vivo, macrophages recovered from the liver were analyzed by flow cytometry on day 2. Microparticle-loaded macrophages (DexP3@MΦ) demonstrated significantly greater M2 polarization (2.5-fold relative to control and 2.2-fold relative to nanoparticle group, Fig. 3H). This was accompanied by the most pronounced reduction in M1 marker expression [3.1-fold lower than the control group and 1.7-fold lower than the nanoparticle group (DexP0.2@MΦ)] (Fig. 3I). At the therapeutic end point, although all diseased mice experienced similar weight loss during the acute inflammatory phase, DexP3@MΦ-treated animals recovered more rapidly, regaining 96.2% of baseline body weight by day 5, compared to 85.3% in the DexP0.2@MΦ group (Fig. 3J). This accelerated recovery correlated with increased anti-inflammatory (Fig. 3K) and reduced proinflammatory cytokine expression (Fig. 3, L to N). Organ indices further reflected therapeutic benefit: DexP3@MΦ-treated mice exhibited normalized lung, liver, and spleen indices (Δ < 8%, *P* > 0.1), comparable to healthy controls. In contrast, untreated [negative control (NC)] and DexP0.2@MΦ groups showed persistent organ swelling (Fig. 3, O to Q). Heart and kidney indices remained unchanged across all groups (Fig. 3, R and S), consistent with heterogeneous organ involvement in the systemic inflammation model (*22*, *23*). In addition, serum levels of liver [alanine aminotransferase (ALT), aspartate aminotransferase (AST), and alkaline phosphatase (ALP)] and kidney [blood urea nitrogen (BUN) and creatinine (Cr)] biomarkers remained within physiological ranges in all treated animals (Δ < 15%, *P* > 0.1), confirming good systemic biosafety (fig. S13).

To determine whether the therapeutic effects observed in the systemic inflammation model arise solely from adoptively transferred macrophages or also involve endogenous immune cells, we performed macrophage depletion experiments using clodronate liposomes (Clo) (fig. S14A). Flow cytometric analysis confirmed efficient macrophage depletion in the spleen following Clo treatment. Adoptive DexP3@MΦ transfer resulted in a modest recovery trend in splenic macrophage proportions compared with Clo alone (fig. S14, B and C), although this did not reach statistical significance due to the limited number of cells infused (2 × 10^6^ per mouse). Compared with DexP3@MΦ treatment alone, macrophage depletion markedly attenuated therapeutic efficacy. Although partial body weight recovery was observed in the DexP3@MΦ + Clo group, recovery was delayed and incomplete relative to the DexP3@MΦ group (fig. S14D). Analysis of organ indices showed no significant differences in heart and kidney across groups, whereas liver, lung, and spleen indices were significantly improved in the DexP3@MΦ group compared with macrophage-depleted animals, consistent with reduced systemic inflammation (fig. S14, E to I). Treatment with Clo alone did not confer therapeutic benefit. Serum cytokine profiling further supported a macrophage-dependent mechanism. DexP3@MΦ therapy significantly reduced proinflammatory cytokines (IL-6, TNF-α, and IL-1β) and enhanced anti-inflammatory cytokines (IL-10), whereas these effects were blunted upon macrophage depletion (fig. S14, J to M). These results indicated that the observed therapeutic effects depend on both adoptively transferred macrophages and the endogenous macrophage compartment.

Collectively, these findings demonstrate that microparticles, by virtue of their prolonged intracellular retention, ensure accurate and durable dosing within macrophages. This sustained intracellular dose drives stable anti-inflammatory polarization and accelerates resolution of systemic inflammation. These results position intracellular retention–mediated dose accuracy as a critical and underexplored design parameter for optimizing cell-mediated drug delivery systems.

### Intracellular microdrugs do not interfere with the chemotactic migration ability of macrophages

**Fig. 4.**
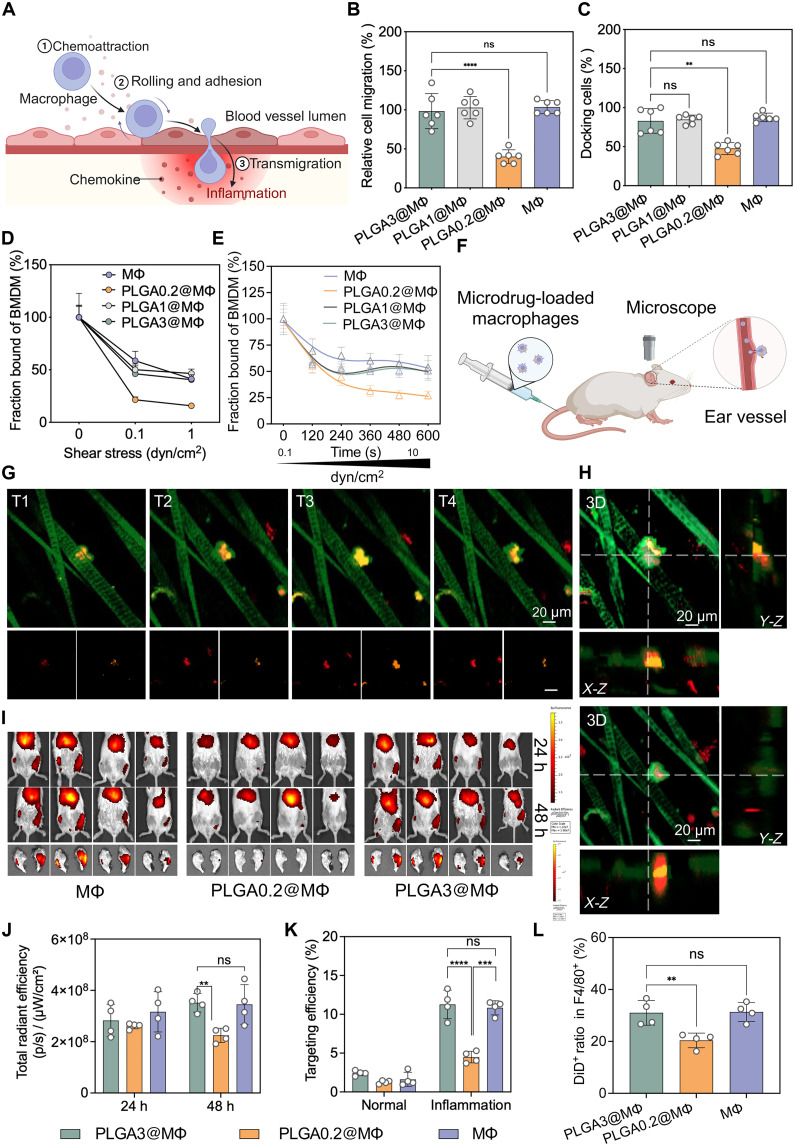
Microparticle-loaded macrophages efficiently adhere to inflamed endothelium and actively transmigrate across the endothelial barrier. (**A**) Schematic illustration of the key steps in the leukocyte adhesion cascade: chemoattraction, rolling, adhesion, and transmigration. Created in BioRender. Huang, Y. (2026) https://BioRender.com/9rdebyp. (**B** and **C**) Transwell chemotaxis (B) and docking (C) efficiency of macrophages (MΦ, PLGA0.2@MΦ, PLGA1@MΦ, and PLGA3@MΦ) (*n* = 6). (**D**) Adhesion dynamics of macrophages (MΦ, PLGA0.2@MΦ, PLGA1@MΦ, and PLGA3@MΦ) under static shear stress (0.1 and 1 dyn/cm^2^) (*n* = 6). (**E**) Adhesion dynamics of macrophages (MΦ, PLGA0.2@MΦ, PLGA1@MΦ, and PLGA3@MΦ) under dynamic shear stress (increase from 0.1 to 10 dyn/cm^2^) (*n* = 6, spline curves were added to illustrate trends). (**F**) Schematic illustration of the experimental workflow for (G). Created in BioRender. Huang, Y. (2026) https://BioRender.com/d6pyein. (**G**) Transendothelial migration of PLGA3@MΦ observed by CLSM (green: Alexa488-CD31, vessel; red: DiD-macrophage; yellow: Rhodamine B-PLGA microparticles; scale bar: 20 μm). (**H**) 3D reconstruction with orthogonal cross-sectional views (*XZ* and *YZ* planes) of PLGA3@MΦ during the initial and final stages of transendothelial migration (green: Alexa488-CD31, vessel; red: DiD-macrophage; yellow: Rhodamine B-PLGA microparticles; scale bar: 20 μm). (**I**) In vivo tracking of adoptively transferred macrophages (MΦ, PLGA0.2@MΦ, and PLGA3@MΦ) in a murine inflamed muscle model using whole-body IVIS imaging at 24 and 48 hours, followed by ex vivo imaging at 48 hours (*n* = 4). (**J**) Quantitative fluorescence intensity of muscle-infiltrated macrophages at 24 and 48 hours (*n* = 4). (**K**) Targeting efficiency of the adoptively transferred macrophages recovered from the muscle (MΦ, PLGA0.2@MΦ, and PLGA3@MΦ) in bilateral skeletal muscle (*n* = 4). (**L**) Proportion of adoptively transferred macrophages among total macrophages (MΦ, PLGA0.2@MΦ, and PLGA3@MΦ) in inflamed muscle (*n* = 4). *P < 0.05, **P < 0.01, and ****P < 0.0001; data are shown as mean ± SD, statistical significance was analyzed using a one-way ANOVA with Tukey’s multiple comparisons [(B) and (J) to (L)], or Brown-Forsythe and Welch ANOVA tests, followed by Dunnett’s T3 multiple comparisons due to unequal variances (C).

Effective leukocyte trafficking requires cells to navigate through narrow vasculature and adhere to inflamed endothelium via the adhesion cascade, which includes rolling, firm adhesion, and transendothelial migration (Fig. 4A) (*24*). We thus investigated whether intracellular drug affects macrophage migration under conditions mimicking physiological barriers.

In transwell assays simulating narrow vascular constraints, microparticle-loaded macrophages exhibited chemotactic migration comparable to unloaded macrophages (MΦ), indicating that internalization of 1- to 3-μm microparticles does not impair directed motility. In contrast, PLGA0.2@MΦ showed over 50% reduction in migration, suggesting size-dependent disruption of migratory capacity (Fig. 4B). We next assessed macrophage adhesion to inflamed endothelium under physiological shear stress. Microparticle-loaded macrophages maintained high adhesion efficiency, similar to MΦ, while PLGA0.2@MΦ showed only half adhesion compared with MΦ (Fig. 4C). Under escalating flow rates, adhesion deficits in the nanoparticle group became more pronounced. At both venous (0.1 to 1 dyn/cm^2^) and arterial (≥5 dyn/cm^2^) shear conditions, microparticle-loaded macrophages maintained robust endothelial interactions, with adhesion levels approximately twice those of the PLGA0.2@MΦ group (Fig. 4, D and E).

Given that firm adhesion is a prerequisite for transendothelial migration, we next evaluated whether intracellular microparticles affect the ability of macrophages to extravasate vasculature at inflamed sites (Fig. 4F). Using intravital microscopy in a mouse ear inflammation model, we tracked DiD-labeled macrophages loaded with Rhodamine B–labeled microparticles. CD31 immunostaining was used to delineate vascular boundaries during intravital imaging (*25*–*27*). Within 6 to 12 hours postinjection, macrophages migrated toward the inflamed vasculature and dynamically engaged with the activated endothelium. Two-dimensional (2D) time-lapse imaging revealed progressive positional change of microparticle-loaded macrophages from initial overlap with the vascular boundary to subsequent spatial separation, suggestive of transendothelial migration (Fig. 4G). To confirm their position relative to the vasculature, we performed 3D reconstructions at early and late time points. Initial 3D reconstructions and orthogonal projections revealed clear colocalization of vessel, macrophage, and microparticle signals in all dimensions, confirming that microparticle-loaded macrophages were initially confined within the vascular lumen. At later time points, the macrophage and its intracellular microparticle cargo were detected beyond the CD31-defined vascular boundary, consistent with extravascular localization after transmigration. Crucially, the colocalization of macrophages and microparticles persisted throughout migration, indicating that microparticles remained intracellular and did not hinder macrophage mobility (Fig. 4H). Macrophage-associated green fluorescence observed during transmigration could be transient in vivo (fig. S15) and was not used as the primary criterion for cell localization. Together, these imaging data demonstrate that microparticle-loaded macrophages undergo transendothelial migration from the vascular lumen into perivascular tissue while maintaining intracellular cargo retention.

Encouraged by these findings, we assessed whether microparticle-loaded macrophages could efficiently home to inflamed tissue in vivo. In a localized muscle inflammation model, whole-body fluorescence imaging revealed that both unloaded MΦ and PLGA3@MΦ efficiently accumulated in the inflamed quadriceps, with signal intensity increasing from 24 to 48 hours, indicative of progressive and sustained recruitment. In contrast, PLGA0.2@MΦ exhibited markedly reduced localization at both time points, suggesting impaired migratory capacity. Minimal signal was detected in the contralateral, noninflamed muscle, confirming inflammation-specific homing (Fig. 4, I and J).

The inflamed muscle tissues were further digested for flow cytometric analysis. Targeting efficiency, defined as the percentage of administered macrophages that homed to and recovered from the inflamed site, reached ∼10% in both MΦ and PLGA3@MΦ groups, compared to ∼5% for the PLGA0.2@MΦ group (Fig. 4K), indicating that nanoparticle loading significantly impairs the migration capacity of carrier macrophages. In addition, transferred macrophages accounted for over 30% of the total macrophages in the inflamed muscle in MΦ and PLGA3@MΦ groups, but only ∼20% in the PLGA0.2@MΦ group (Fig. 4L), reflecting a reduced representation within the tissue-resident macrophage pool.

Collectively, these results demonstrate that under matched intracellular loading, drug size emerges as the critical determinant of whether carrier cells maintain their migratory capacity. Intracellular microparticles do not impair the chemotactic migration of carrier cells to inflamed sites, while nanoparticle-loaded macrophages exhibited substantially reduced recruitment.

### Nanodrugs impair macrophage migration by disrupting cytoskeletal homeostasis via intracellular ROS accumulation

**Fig. 5.**
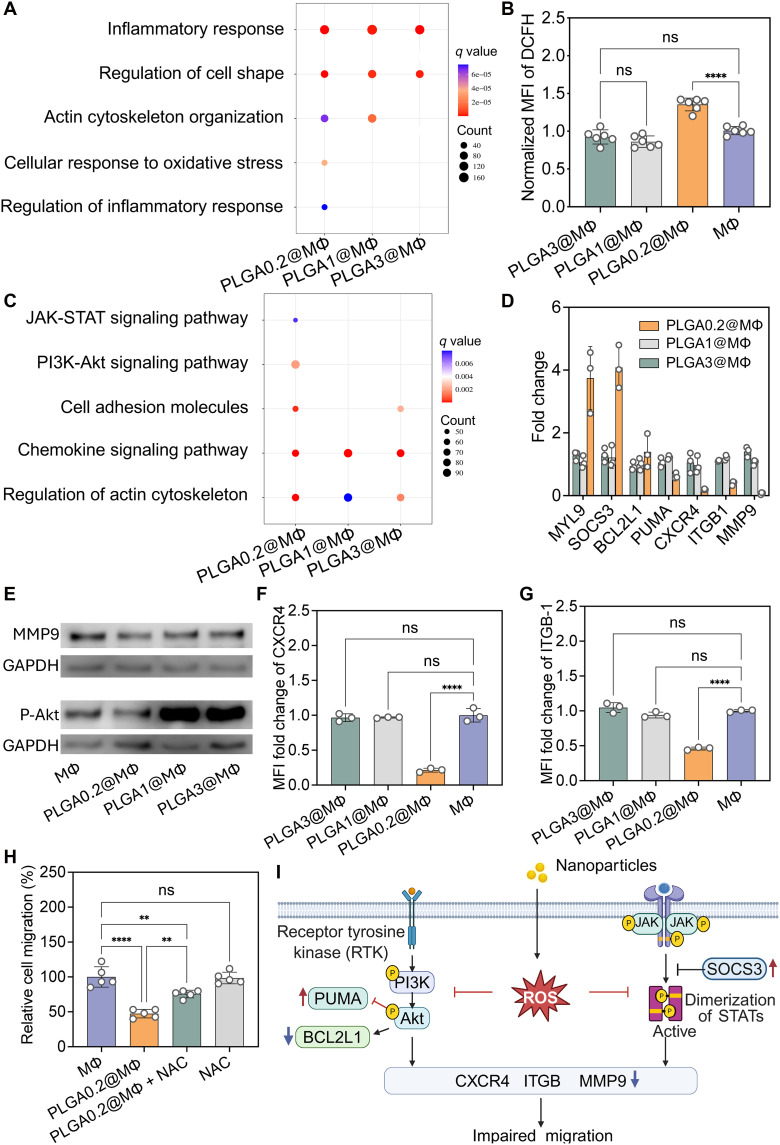
Intracellular nanoparticle-induced ROS accumulation impairs macrophage migration through JAK-STAT and PI3K-Akt pathway suppression. (**A**) GO enrichment analysis (*q* < 0.0001) of differentially expressed genes in particle-loaded macrophages (PLGA0.2@MΦ, PLGA1@MΦ, and PLGA3@MΦ), each compared to untreated macrophages (MΦ). (**B**) Quantification of intracellular ROS by mean fluorescence intensity (MFI) in particle-loaded macrophages (PLGA0.2@MΦ, PLGA1@MΦ, and PLGA3@MΦ), normalized to untreated macrophages (MΦ) (*n* = 6). (**C**) KEGG pathway enrichment analysis (*q* < 0.01) of differentially expressed genes in particle-loaded macrophages (PLGA0.2@MΦ, PLGA1@MΦ, and PLGA3@MΦ), each compared to untreated macrophages (MΦ). (**D**) Quantitative PCR analysis of migration- and cytoskeleton-related genes, including SOCS3, PUMA, BCL2L1, CXCR4, ITGB1, MMP9, and MYL9. (**E**) Representative Western blot images of MMP9 and phosphorylated Akt (P-Akt). (**F** and **G**) Relative expression of CXCR4 (F) and ITGB1 (G) (flow cytometry, *n* = 3). (**H**) Chemotactic migration of macrophages in the presence of NAC (*n* = 5). (**I**) Schematic illustration of the mechanisms by which nanoparticles impair the migratory capacity of carrier macrophages. Created in BioRender. Huang, Y. (2026) https://BioRender.com/8j9iroe. **P < 0.01 and ****P < 0.0001; the data are shown as mean ± SD, statistical significance was analyzed using a one-way ANOVA with Tukey’s multiple comparisons [(B) and (F) to (H)].

To investigate the mechanism by which nanoparticles impair macrophage migration, we performed bulk RNA-sequencing (RNA-seq) on macrophages following an 8-hour efflux period after loading with 0.2-, 1-, or 3-μm PLGA particles under equivalent intracellular payloads. Gene ontology (GO) enrichment analysis revealed that nanoparticle exposure primarily altered gene sets associated with oxidative stress, inflammatory responses, and actin cytoskeleton organization (Fig. 5A).

Guided by these transcriptomic signatures, we next assessed whether oxidative stress and cytoskeletal remodeling were indeed induced at the cellular level. Direct quantification of intracellular reactive oxygen species (ROS) showed a pronounced increase in nanoparticle-loaded macrophages, whereas macrophages exposed to microparticles maintained baseline ROS level (Fig. 5B), identifying oxidative stress as a response specific to nanoparticle internalization. Consistently, staining of filamentous actin (F-actin) with phalloidin revealed enhanced actin polymerization in nanoparticle-loaded macrophages (fig. S16), indicative of increased contractility and disrupted cytoskeletal homeostasis.

To further define the downstream signaling pathways linking these alterations to impaired migration, we conducted Kyoto Encyclopedia of Genes and Genomes (KEGG) pathway analysis. Nanoparticle exposure prominently affected the Janus kinase (JAK)–signal transducers and activators of transcription (STAT) and phosphatidylinositol 3-kinase (PI3K)–Akt signaling pathways, as well as cell adhesion–related pathways, all of which are central regulators of cell motility (Fig. 5C). Quantitative polymerase chain reaction (PCR) confirmed coordinated transcriptional changes within these pathways. The transcriptional suppressor SOCS3 (*28*), a known inhibitor of JAK-STAT signaling, was up-regulated 4.1-fold. In parallel, the proapoptotic gene PUMA was elevated, while the anti-apoptotic effector BCL2L1 was suppressed, indicating PI3K-Akt pathway inhibition (*29*, *30*). Critically, nanoparticle treatment induced a broad down-regulation of genes governing cell adhesion and migration. CXCR4, a chemokine receptor essential for directional migration (*31*), was reduced by 80%; ITGB1 (integrin β1), vital for extracellular matrix adhesion (*32*), declined by 60%; and MMP9, a matrix metalloproteinase required for extracellular remodeling (*33*), decreased by 92.7%. Meanwhile, MYL9, encoding myosin light chain 9 and linked to contractile force generation (*34*), was up-regulated 3.74-fold, reflecting elevated cytoskeletal tension (Fig. 5D and fig. S17). These transcriptomic alterations were further validated at the protein level. Western blotting demonstrated decreased Akt phosphorylation and reduced MMP9 protein levels (Fig. 5E and fig. S18), while flow cytometric analysis confirmed reduced surface expression of CXCR4 and ITGB1 in PLGA0.2@MΦ (Fig. 5, F and G), in agreement with the RNA-seq data.

Last, to determine whether ROS plays a causal role in nanoparticle-induced migratory defects rather than representing a correlative stress response, we performed a loss-of-function experiment using the ROS scavenger *N*-acetylcysteine (NAC). Treatment with 1 mM NAC effectively reduced intracellular ROS in nanoparticle-loaded macrophages to near-baseline levels (fig. S19A). Flow cytometric analysis showed that NAC treatment substantially, though not completely, restored surface expression of CXCR4 and ITGB1 in nanoparticle-loaded macrophages, while exerting minimal effects on unloaded control cells (fig. S19, B and C). Consistent with these molecular changes, NAC partially rescued the impaired chemotactic migration of nanoparticle-loaded macrophages in transwell assays, again without affecting baseline migration in control macrophages (Fig. 5H). Therefore, ROS is a key functional mediator linking nanoparticle uptake to disruption of migratory signaling and cytoskeletal regulation in macrophages.

Together, these results identify nanoparticle internalization as a trigger for ROS generation, which in turn disrupts key migratory signaling pathways, suppresses adhesion machinery, and destabilizes actin dynamics. The convergence of these molecular perturbations ultimately culminates in impaired macrophage chemotaxis.

### Efficient macrophage homing enables faithful drug delivery and inflammation resolution

**Fig. 6.**
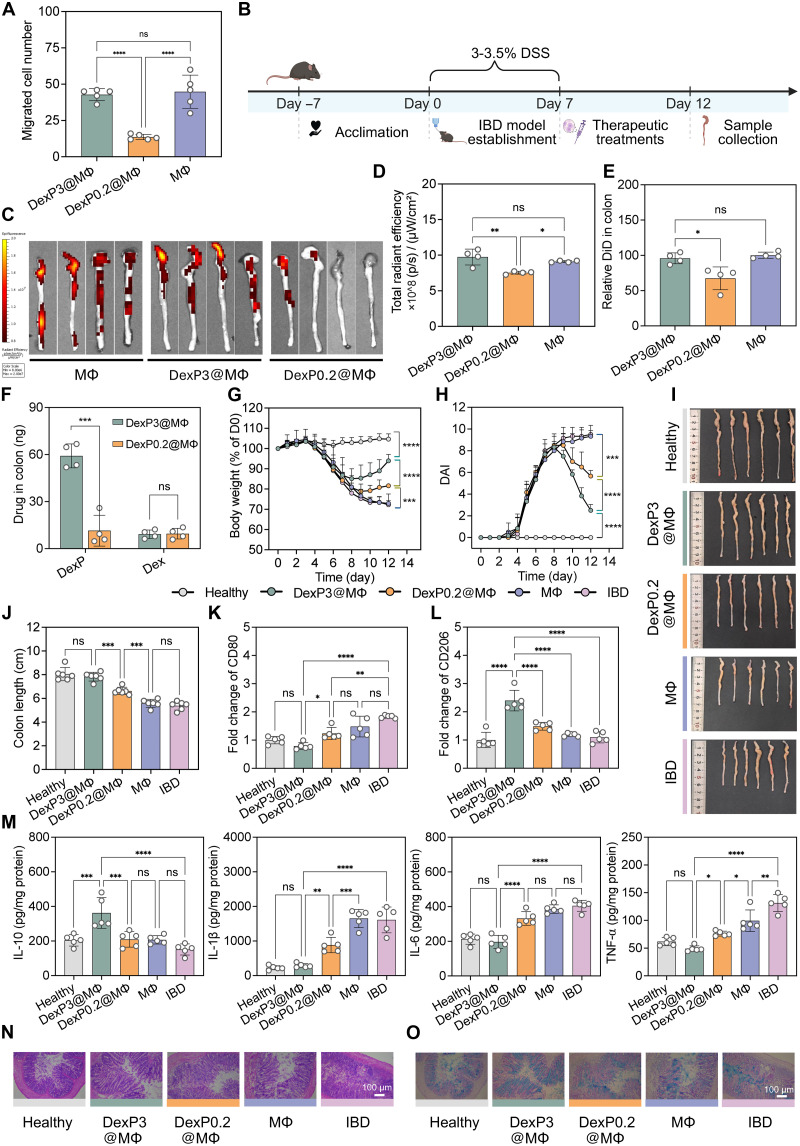
Anti-inflammatory microparticle-engineered macrophages migrate to the inflamed colon and ameliorate IBD. (**A**) Transwell-based chemotaxis of macrophages (MΦ, DexP0.2@MΦ, and DexP3@MΦ) (*n* = 5). (**B**) Schematic illustration of adoptive macrophage therapy for IBD. Created in BioRender. Huang, Y. (2026) https://BioRender.com/st725a2. (**C** and **D**) IVIS imaging (C) and corresponding quantification of fluorescence intensity (D) of adoptively transferred macrophages (MΦ, DexP0.2@MΦ, and DexP3@MΦ) in the inflamed colons (*n* = 4). (**E** and **F**) Quantification of macrophage infiltration into the colon based on DiD fluorescence (E), and corresponding colonic amount of cell-delivered DexP and Dex by LC-MS at 24 hours (F) (*n* = 4). (**G**) Body weight changes over the treatment course (*n* = 6). (**H**) DAI scoring over time (*n* = 6). (**I** and **J**) Colon images (I) and colon length (J) (*n* = 6). (**K** and **L**) Flow cytometry analysis of colonic macrophages: proinflammatory CD80^+^ (K) and anti-inflammatory CD206^+^ subsets (L) (*n* = 5). (**M**) Cytokine levels in colon homogenates, normalized to total protein content (*n* = 5). (**N** and **O**) Histopathological analysis: (N) H&E staining (inflammatory infiltrate scoring) and (O) Alcian Blue (AB) staining for goblet cell preservation (scale bars, 100 μm). *P < 0.05, **P < 0.01, ***P < 0.001, and ****P < 0.0001; the data are shown as mean ± SD, statistical significance was analyzed using a one-way ANOVA with Tukey’s multiple comparisons [(A), (D), (E), (G), (H), and (J) to (M)] or a two-tailed unpaired Student’s *t* test (F).

To determine whether the migration advantages conferred by microparticle loading translate into therapeutic benefits, we first assessed functional motility using transwell assays. Macrophages loaded with DexP nanoparticles exhibited markedly reduced migration, while those carrying DexP microparticles retained normal motility (Fig. 6A), recapitulating the size-dependent migration effects previously observed with PLGA particles (Fig. 4B).

We next evaluated therapeutic performance in a dextran sulfate sodium (DSS)–induced inflammatory bowel disease (IBD) model (Fig. 6B). After 7 days of DSS exposure, equal doses of drug-loaded macrophages were intravenously administered. Twenty-four hours postinjection, ex vivo fluorescence imaging revealed preferential accumulation of DexP3@MΦ in the inflamed colon, at levels comparable to MΦ group and ∼30% higher than the DexP0.2@MΦ group (Fig. 6, C and D). This differential accumulation was further validated by quantitative fluorescence analysis of colon homogenates, which showed a 33.3% lower signal in the DexP0.2@MΦ group (Fig. 6E). These biodistribution differences directly affected drug delivery: liquid chromatography–mass spectrometry (LC-MS) quantification revealed an 8.64-fold higher amount of DexP in colonic tissue in the DexP3@MΦ group compared to the DexP0.2@MΦ group (Fig. 6F). However, no significant difference in bulk free Dex was observed between these two groups at the analyzed time point (Fig. 6F). This may be because the bulk free Dex level at 24 hours likely reflects a slow, sustained drug release by enzyme-mediated hydrolysis and the rapid redistribution/clearance of liberated Dex, thereby blunting differences in tissue-averaged free Dex despite markedly different DexP depots.

Treatment outcomes aligned with these biodistribution patterns. While untreated and blank macrophage-treated mice experienced progressive weight loss, animals receiving DexP3@MΦ treatment showed substantial recovery, regaining over 90% of baseline weight by day 12. In comparison, the DexP0.2@MΦ group recovered to only ∼80% (Fig. 6G). Disease activity index (DAI) scores followed similar trends, with DexP3@MΦ producing the greatest improvement (Fig. 6H). Colon length, a sensitive marker of colitis severity, was restored to near-healthy levels in the DexP3@MΦ group, while partially recovered (82.4%) in the DexP0.2@MΦ group (Fig. 6, I and J). Flow cytometry revealed a shift toward an anti-inflammatory phenotype in the DexP3@MΦ group, with increased CD206 (2.32-fold) and reduced CD80 expression (15%) (Fig. 6, K and L, and fig. S20), as further supported by colon immunofluorescence staining (fig. S21). This was accompanied by elevated anti-inflammatory cytokine (IL-10) production and decreased levels of proinflammatory cytokines (IL-1β, IL-6, and TNF-α), indicating immunomodulatory activity (Fig. 6M). Histological analysis reinforced these outcomes. Mice treated with DexP3@MΦ showed improved epithelial integrity, reduced immune cell infiltration, and preserved goblet cell morphology, in contrast to severe tissue damage observed in untreated and blank MΦ groups (Fig. 6, N and O).

To further determine whether the therapeutic benefit required macrophage-mediated delivery rather than passive drug release alone, we compared DexP3@MΦ with 3-μm DexP particles administered at an equivalent DexP dose in the DSS-induced IBD model. This comparison served as an important supporting control to distinguish cell-mediated delivery from particle-only treatment. While DexP particles alone produced only modest improvements in clinical score, colon length, and histological inflammation, DexP3@MΦ treatment resulted in significantly greater attenuation of colitis across all end points (fig. S22). These data further support that the therapeutic benefit cannot be explained by passive administration of DexP particles alone, but instead depends on macrophage-mediated transport and delivery in vivo.

Together, these results demonstrate that preserving macrophage homing is essential for accurate drug delivery to inflamed tissues and for achieving effective resolution of inflammation. This establishes cellular migration capacity as a critical determinant of therapeutic success in macrophage-based drug delivery systems.

## DISCUSSION

CDDS represent a promising therapeutic modality for treating chronic inflammation and autoimmune diseases (*35*–*37*). However, a central uncertainty persists: Drugs successfully loaded into carrier cells in vitro do not necessarily translate into effective doses in vivo. This disconnect between loading and delivery has long been overlooked, largely because most studies rely on uptake efficiency as a proxy for delivery performance, while lacking systematic analyses that bridge in vitro loading with in vivo outcomes (*38*). Our study directly addresses this gap by performing a systematically controlled comparison in which common drug formulations, nanoparticles versus microparticles, served as a probe to examine intracellular fate, migration, and therapeutic performance under equivalent intracellular payloads. This framework enabled us to determine not only how drug properties influence delivery, but more importantly, whether in vitro loading reliably translates into accurate in vivo dosing.

First, we quantitatively established that retention in vitro is not a reliable predictor of stability in vivo. While nearly 60% of nanoparticles appeared retained under in vitro conditions, our in vivo tracking revealed amplified losses, with 53.8% of nanoparticles being expelled by adoptive macrophages in the lung and up to 83.2% of nanoparticles being lost upon hepatic arrival. This in vivo–in vitro gap likely arises from physiological factors absent in static culture, most notably systemic dilution (*39*) coupled with organ-specific phagocytic sink effects (*40*), hemodynamic stress (*41*, *42*), and extracellular matrix resistance (*43*–*45*), which collectively sustain steep extracellular gradients and promote vesicle turnover, thereby magnifying exocytosis and eroding intracellular dose accuracy. The accelerated efflux in the liver relative to the lung likely reflects a more favorable “sink condition” created by systemic dilution during transit from lung to liver (*39*) and by the highly active endocytic-exocytic niche formed by liver sinusoidal endothelial cells and Kupffer cells (*46*, *47*). These results underscore that the intracellular drug loading amount in vitro does not equal the effective dose that can be maintained within cells under physiological circulation. In other words, the intracellular drug content estimated in vitro represents an overestimation of the dose that can be faithfully delivered in vivo. By contrast, macrophages carrying microparticles preserved their intracellular payloads for at least 7 days in vitro, consistent with a prior study (*48*). In line with this concept, strategies such as encapsulating nanoparticles within microparticles have been previously explored to improve intracellular persistence (*49*). Our findings extend these efforts by indicating that microparticle-loaded macrophages maintained delivery competence even under systemic exposure in vivo.

To probe this divergence, we confirmed that nanoparticles were primarily exocytosed via lysosome-mediated pathways, consistent with previous studies (*47*, *50*). However, the underlying basis for the sustained intracellular retention of microparticles remains less defined. Prior studies of intracellular pathogens suggest that escape from host cells is an active process in which pathogen-secreted factors perturb intracellular membranes and trigger host cytoskeletal remodeling (*51*–*53*); notably, such behavior is greatly diminished for nonviable pathogens or inert particles (*54*, *55*). In line with this distinction, the microparticles in our study remained confined within phagolysosomal compartments (Figs. 2E and 3D) and showed no observable sign of actin-associated intracellular mobilization (Fig. 2F). Such biological immobility may contribute to their prolonged intracellular persistence, ensuring that what is loaded in vitro remains available for delivery in vivo*.* Together, these findings define intracellular persistence, previously unquantified across in vitro and in vivo contexts, as a governing principle of delivery fidelity in CDDS.

We next investigated whether intracellular drug loading interferes with carrier cell migration, another prerequisite for delivery fidelity. Unlike previous studies that compared the cell behavior under equal incubation concentration or time (*56*, *57*), thus conflating uptake kinetics with functional outcomes, we normalized intracellular drug payloads to directly assess how nano- versus microdrugs influence migration under equivalent delivery conditions. This approach reflects the true requirement of cell-based drug delivery, namely, that the effective intracellular dose, rather than incubation time, determines whether drugs can be faithfully transported to diseased sites.

Under these controlled conditions, nanoparticle-loaded macrophages displayed compromised migration. Transcriptomic profiling revealed that nanoparticles triggered oxidative stress and disrupted multiple signaling pathways essential for migration, including JAK-STAT and PI3K-Akt. These disruptions led to elevated cytoskeletal contractility, down-regulation of key adhesion molecules (such as CXCR4 and ITGB1), and destabilized actin dynamics. Consistently, scavenging intracellular ROS partially alleviated the migration defect, indicating that oxidative stress acts as a functional mediator rather than a passive correlate. These findings reframe the long-held assumption that nanoparticles are universally migration-compatible and instead identify cellular stress and signaling reprogramming as key drivers of impaired chemotaxis. On the other hand, macrophages loaded with spherical, monodisperse microparticles preserved chemotaxis and efficiently infiltrated inflamed tissues while stably retaining their intracellular cargo.

Through intravital imaging, we further visualized and reconstructed three-dimensional spatiotemporal trajectories of microparticle-loaded macrophages crossing vascular endothelium, providing dynamic in vivo evidence that is not available in previous static end point tissue sections (*58*, *59*). This observation addresses a long-standing gap and confirms that large, fully internalized microparticles do not interfere with cellular trafficking across vascular barriers, supporting their compatibility with systemic delivery. While earlier work using anisotropic micro-sized cargos (e.g., discoidal multistage vectors, 3 μm in-plane and 600 nm in thickness) reported minimal migration defects (*60*), such geometries may introduce directional mechanical bias (*61*, *62*) that limits generalizability. Our isotropic microparticles minimize cytoskeletal interference and preserve cell deformability during transmigration.

Therapeutic studies further validated our findings. Despite equivalent intracellular drug dose, microparticle-loaded macrophages exhibited superior therapeutic efficacy across two complementary models. In the systemic inflammation model, stable intracellular retention sustained an anti-inflammatory phenotype under inflammatory stress, demonstrating the importance of preventing premature exocytosis. In the IBD model, preserved migratory capacity enabled efficient homing to inflamed lesions, confirming that trafficking fidelity dictates local therapeutic efficacy. Once delivered to target tissues, adoptively transferred macrophages are not passive drug depots but active immunomodulatory agents that can reprogram resident macrophage populations and reshape the local immune microenvironment, a mechanism that has been widely implicated in macrophage-based therapies across inflammatory, fibrotic (*48*), tumor-associated settings (*63*). Consistent with this interpretation, comparison with free DexP microparticles in the DSS model further showed that particle administration alone could not recapitulate the full therapeutic benefit of DexP3@MΦ, underscoring the functional importance of macrophage-mediated delivery beyond the drug formulation itself (fig. S22).

In summary, our study demonstrates that the long-standing disconnect between in vitro loading and in vivo delivery arises from two indispensable determinants: intracellular drug retention and carrier cell migration. By ensuring that drugs remain available within carrier cells and that these cells retain their capacity for active homing, microdrug engineering provides a “load once, deliver fully” strategy that enables accurate intracellular dosing, preserved homing, and superior therapeutic efficacy.

More broadly, our work calls for a re-evaluation of drug design principles in CDDS, moving beyond uptake efficiency to encompass intracellular fate and functional preservation. By elucidating how drug size governs intracellular trafficking and functional outcomes, our study expands the design space of CDDS and contributes a robust, mechanism-driven foundation for engineering immune cells with programmable therapeutic behaviors. As the field advances toward more refined and systemically delivered cellular interventions, such strategies will be critical in advancing the precision, safety, and versatility of CDDS.

## MATERIALS AND METHODS

### Cell lines and animals

Murine bEnd.3 cells were cultured in Dulbecco’s modified Eagle’s medium supplemented with 10% fetal bovine serum (FBS) at 37°C in a humidified 5% CO_2_ incubator. Female BALB/c and male C57BL/6 mice were purchased from the Hubei Provincial Center for Disease Control and Prevention and Jiangsu Huachuang Xinnuo Pharmaceutical Technology Company. Animals were housed under specific-pathogen-free conditions (22°C, 12-hour light/dark cycle) with ad libitum access to food and water. All animal experiments were performed in compliance with the Guide for the Care and Use of Laboratory Animals and approved by the Animal Ethics Committee of Huazhong University of Science and Technology (IACUC number: HUST-IACUC-2025-0040).

### Isolation of BMDMs

Bone marrow was flushed from the femurs and tibias of 6- to 8-week-old mice using PBS and a sterile syringe. The suspension was passed through a 70-μm cell strainer, followed by red blood cell lysis using lysis buffer. Cells were washed with PBS and cultured in RPMI 1640 medium supplemented with 10% FBS and macrophage colony-stimulating factor (30 ng/ml; M-CSF, PeproTech, catalog no. 315-02) for 6 days to induce macrophage differentiation. Adherent cells were harvested, and viability was assessed by trypan blue exclusion.

### Particle preparation and characterization

DexP was synthesized based on previously reported protocols. PLGA and DexP microparticles were fabricated using a custom-designed flow-focusing microfluidic device. The organic phase consisted of PLGA (10 mg/ml, PURAC 5002A, the Netherlands) or DexP (10 mg/ml) dissolved in ethyl acetate, while the aqueous phase contained 1% (w/v) Poloxamer 407 (BASF, pH 7.4). PLGA and DexP nanoparticles were fabricated using a co-flow microfluidic device. The organic phase contained 10 mg/ml of either PLGA (in acetone) or DexP (in acetonitrile), while the aqueous phase contained 1% (w/v) Poloxamer 407 (BASF, pH 7.4; supplemented with 10% MgCl_2_ for DexP nanoparticle fabrication). Particle size was controlled by adjusting the flow rates of the two phases. Particles were collected, washed three times with Milli-Q water, and stored at 4°C. Fluorescent PLGA particles were prepared similarly, with the addition of 1% PLGA–Rhodamine B (908622, Sigma-Aldrich) to the organic phase. For in vivo exocytosis assessment, the percent of PLGA–Rhodamine B was increased to 5%.

Particle size and morphology were analyzed by scanning electron microscopy (SEM; JSM-IT800, JEOL, Japan) or transmission electron microscopy (TEM; Tecnai F12, FEI, USA). Hydrodynamic diameter and PDI of nanoparticles were determined using dynamic light scattering (Zetasizer Nano-ZS, Malvern, UK).

### Construction and characterization of particle-loaded macrophages

To achieve comparable intracellular particle mass or drug content across different particle sizes, macrophages were incubated with PLGA particles (0.2, 1, and 3 μm) or DexP particles (0.2 and 3 μm) at varying concentrations for a fixed loading period of 4 hours in complete culture medium. Cells were washed with PBS to remove noninternalized particles. Intracellular loading was subsequently quantified as described below, and the particle concentrations that resulted in equivalent intracellular PLGA mass or DexP content per cell were identified. These optimized loading conditions were then used consistently for all subsequent in vitro assays and in vivo adoptive transfer experiments unless otherwise stated.

For quantification of intracellular PLGA particle loading, rhodamine B–labeled PLGA particles were used. After particle incubation and thorough washing to remove noninternalized particles, macrophages were lysed, and the fluorescence intensity of the lysates was measured using a microplate reader. A calibration curve was generated by adding known amounts of rhodamine-labeled PLGA particles into macrophage lysates from untreated macrophages and plotting fluorescence intensity against PLGA mass. Using this calibration, fluorescence signals from particle-loaded cell lysates were converted to intracellular PLGA mass, enabling calculation of the average PLGA mass per cell.

For DexP particles, intracellular loading was quantified by high-performance liquid chromatography (HPLC; Agilent, 1260 infinity) analysis of cell lysates. After particle incubation and washing, cells were lysed, and DexP was extracted and quantified using an external standard calibration curve. Chromatographic separation was performed on an Agilent Poroshell 120 EC-C18 column (4.6 mm × 150 mm, 4-μm particle size) using an HPLC system equipped with a ultraviolet (UV)–visible detector. The mobile phase consisted of acetonitrile-isopropanol (85:15, v/v). The flow rate was maintained at 1.0 ml/min, the detection wavelength was set to 239 nm, the injection volume was 20 μl, and the column temperature was precisely controlled at 30°C.

Cell viability was evaluated using the CCK-8 assay. Intracellular localization of PLGA particles was assessed by LysoTracker Green (Invitrogen, catalog no. L7526) staining, followed by imaging under confocal laser scanning microscopy (CLSM; Carl Zeiss Microscopy GmbH, Oberkochen, Germany).

### In vitro exocytosis assays

Macrophages were seeded with ∼50% of density in plates and incubated with particles for 4 hours, after which the particle-loaded macrophages were further incubated in fresh culture medium for 1, 3, 6, 10, or 24 hours. The medium was replaced every 1 to 2 hours to remove the exocytosed particles. At the indicated time points, cells were harvested and analyzed for intracellular particle content using flow cytometry (CytoFLEX, BECKMAN) for fluorescent PLGA particles, or HPLC for DexP particles.

Exocytosis pathways were investigated by treating cells with pharmacological inhibitors, including brefeldin A (50 μM, MedChemExpress, catalog no. HY-16592) and bafilomycin A1 (0.25 μM, MedChemExpress, catalog no. HY-100558), during the incubation period. The inhibitor-containing medium was refreshed every 1 to 2 hours.

### In vivo distribution and exocytosis assessment

DiD-labeled macrophages loaded with either 0.2- or 3-μm rhodamine B–labeled PLGA particles (2 × 10^6^ cells per mouse, 100 μl) were administered via tail vein injection within 30 min of cell collection (*n* = 5 per group). The cell density, injection volume, and time interval between cell collection and reinfusion were maintained consistently across all subsequent in vivo adoptive transfer experiments unless otherwise stated.

At day 1 and day 5, ex vivo fluorescence imaging was performed using an IVIS to assess cell and particle distribution without cardiac perfusion, so that both intravascularly retained and tissue-associated transferred macrophages could be captured. Lungs and livers were harvested for further analysis by CLSM and flow cytometry.

### DexP drug release

In vitro enzyme-responsive drug release of DexP particles (0.2 and 3 μm) was evaluated in PBS (pH 7.4, 37°C, 100 rpm) with or without lipase (100 U/ml; L798830, Macklin). At designated time points, supernatants were collected by centrifugation and analyzed by HPLC to quantify released dexamethasone. Chromatographic separation was performed on an Agilent Poroshell 120 EC-C18 column (4.6 mm × 150 mm, 4-μm particle size) using an HPLC system equipped with a UV-visible detector. The mobile phase consisted of acetonitrile–0.5% glacial acetic acid aqueous solution (50:50, v/v). The flow rate was maintained at 1.0 ml/min, the detection wavelength was set to 236 nm, the injection volume was 20 μl, and the column temperature was precisely controlled at 30°C.

### Macrophage polarization and anti-inflammatory assays

Macrophages were seeded into 12-well plates (1 × 10^4^ cells per well) and divided into seven groups: control, LPS (100 ng/ml), IL-4 (20 ng/ml), DexP0.2@MΦ, DexP3@MΦ, DexP0.2@MΦ + LPS, and DexP3@MΦ + LPS. Cells were incubated for 1, 3, or 5 days, after which culture media were collected for enzyme-linked immunosorbent assay (ELISA), and cells were stained with fluorescein isothiocyanate (FITC)–anti-F4/80 (BioLegend, catalog no. 123108, clone BM8, lot no. B434263), PE–anti-CD206 (BioLegend, catalog no. 141706, clone C068C2, lot no. B427416), and APC–anti-CD80 (BioLegend, catalog no. 104714, clone 16-10A1, lot no. B445981). Fc blocker (Starter, catalog no. SOB0599) was used for Fc blocking. Analysis was performed with FlowJo (10.8.1).

### Systemic inflammation model and treatment

Systemic inflammation was induced in female BALB/c mice (6 to 8 weeks old) via intraperitoneal injection of LPS (*Escherichia coli* O55:B5, MedChemExpress, catalog no. HY-D1056): 15 mg/kg on day 0, followed by 5 mg/kg on day 2, unless otherwise specified. For treatment groups, 2 × 10^6^ cells were administered via tail vein injection 1 hour after the initial LPS challenge.

#### Early in vivo macrophage polarization

For early mechanistic analysis, mice were randomly assigned to three groups: (A) macrophages (MΦ), (B) DexP-loaded macrophages with 0.2-μm particles (DexP0.2@MΦ), and (C) DexP-loaded macrophages with 3-μm particles (DexP3@MΦ). In this cohort, mice were euthanized on day 2 without the second LPS injection. Livers were harvested and processed into single-cell suspensions. Adoptively transferred macrophages were identified based on DiD fluorescence, followed by Fc blocking and phenotypic analysis using PE–anti-CD206 and PE/cyanine7-anti-CD80 (BioLegend, catalog no. 104734, clone 16-10A1, lot no. B413526) by flow cytometry.

#### Therapeutic evaluation

For therapeutic evaluation, mice were randomly assigned to five groups: (A) Healthy control, (B) LPS only (NC), (C) macrophages (MΦ), (D) DexP-loaded macrophages with 0.2-μm particles (DexP0.2@MΦ), and (E) DexP-loaded macrophages with 3-μm particles (DexP3@MΦ). Body weight was monitored daily throughout the study. At the experimental end point (day 5), blood and major organs were harvested for biochemical analysis, cytokine profiling, and assessment of the organ index.

#### Macrophage depletion experiment

For the macrophage depletion experiment, mice were randomly assigned to four groups: (A) LPS only (NC), (B) Clodronate liposomes (Clo, Yasen), (C) DexP3@MΦ + Clo, and (D) DexP3@MΦ. Clo was injected intraperitoneally into mice 24 hours before macrophage injection (100 μl/10 g body weight, 5 mg/ml). To verify macrophage depletion, spleens were harvested from mice at the designated time point and processed into single-cell suspensions for flow cytometric analysis. After Fc blocking, cells were stained with antibodies against APC–anti-CD45 (BioLegend, catalog no. 103112, clone 30-F11, lot no. B417515) and FITC–anti-F4/80, and splenic macrophages were identified as CD45^+^F4/80^+^ cells. The proportion of CD45^+^F4/80^+^ macrophages was quantified to assess the efficiency of clodronate-mediated macrophage depletion. Body weight was monitored daily throughout the study. At the experimental end point, blood and major organs were harvested for biochemical analysis, cytokine profiling, and assessment of the organ index.

### Macrophage migration assays

(i) Transwell migration: 8 × 10^4^ macrophages were seeded in the upper chamber (8-μm pore, Corning) and cocultured with LPS-stimulated BMDMs (100 ng/ml, 12 hours) in the lower chamber. After 24 hours, nonmigrated cells were removed. Migrated cells were fixed with 4% paraformaldehyde (PFA), stained with 4′,6-diamidino-2-phenylindole (DAPI), and visualized using CLSM.

(ii) Docking: For static docking assays, bEnd.3 cells were seeded into four-chamber LabTek glass slides and cultured overnight to allow adherence. Cells were then stimulated with TNF-α (50 ng/ml) for 18 hours to induce endothelial activation. Subsequently, 8 × 10^4^ DiD-labeled macrophages (various formulations) were added to each chamber and allowed to interact for 60 min under static conditions. Nonadherent cells were gently removed by PBS washing, and slides were fixed with 4% PFA for 10 min at room temperature. Adherent cells were counted from randomly selected fields of view using CLSM.

For dynamic docking assays, bEnd.3 cells were seeded into Ibidi μ-Slide I^0.4^ Luer (Ibidi-treated) flow chambers at a density sufficient to form a confluent monolayer overnight, followed by stimulation with TNF-α (50 ng/ml) for 18 hours. DiD-labeled macrophages (1 × 10^6^ cells/ml) were added to the slides and incubated for 20 min under static conditions to allow initial adhesion. Slides were then subjected to a constant shear stress of 0.5 dyn/cm^2^ for 10 min using a peristaltic pump-based flow system. Confocal images were acquired before and after shear exposure, and macrophage retention was quantified from randomly selected fields of view.

(iii) Adhesion under shear stress: For fixed shear stress assays, bEnd.3 cells were seeded into Ibidi μ-Slide I^0.4^ Luer flow chambers, cultured overnight, and stimulated with TNF-α (50 ng/ml) for 18 hours. DiD-labeled macrophages (1 × 10^6^ cells/ml) were added and allowed to adhere for 5 min under static conditions. Chambers were then exposed to predefined shear stresses of 0.1 or 1 dyn/cm^2^, each for 5 min using a controlled perfusion system. CLSM was used to image the adherent macrophages before and after shear stress exposure.

For varying shear stress assays, the setup was identical to that of the fixed shear experiments. After 5 min of static adhesion, slides were exposed to a sequential increase in shear stress: 0.1, 0.5, 1, 5, and 10 dyn/cm^2^, each maintained for 120 s. After each shear stress level, images were acquired, and retained macrophages were counted from randomly selected fields of view.

(iv) Transendothelial migration: bEnd.3 cells were seeded on 8-μm transwells (24-well plate). After TNF-α stimulation, 4 × 10^4^ DiD-labeled macrophages were added to the upper chamber. After 24 hours, nonmigrated cells were removed, migrated cells were fixed, stained with DAPI, and imaged using CLSM.

### In vivo transmigration

A tetradecanoylphorbol acetate (TPA)–induced ear inflammation model was used to dynamically monitor the extravasation of microparticle-loaded macrophages. In brief, TPA (0.2 mg/ml; dissolved in acetone) was evenly applied to both the inner and outer surfaces of the mouse ear to induce local inflammation. After 12 hours, the ear vasculature was labeled via intravenous injection of Alexa Fluor 488–conjugated anti-CD31 antibody to visualize endothelial structures. At 2-hours postantibody injection, DiD-labeled PLGA3@MΦ were administered via tail vein injection. Subsequently, at 6 to 12 hours postcell administration, the inflamed ear was gently placed between sterile water–moistened cover glasses for imaging. Real-time imaging was performed using a Zeiss LSM980 inverted confocal laser scanning microscope, equipped with a 10× objective and operated using ZEN Blue software (version 3.5). *Z*-stack images were acquired at 1.5-μm intervals to capture three-dimensional cell localization and vessel interaction. Image processing and quantitative analysis were conducted using the same software.

### In vivo targeting efficiency

An acute skeletal muscle inflammation model was established to assess the targeting efficiency of macrophage-based delivery systems. In brief, 100 μl of LPS (1 mg/ml) was intramuscularly injected into the left quadriceps femoris to induce localized inflammation, while the contralateral (right) muscle received no injection and served as an internal control. DiD-labeled macrophages (MΦ), 0.2-μm PLGA-loaded macrophages (PLGA0.2@MΦ), and 3-μm PLGA-loaded macrophages (PLGA3@MΦ) were intravenously administered via the tail vein at equal cell numbers (*n* = 4 per group). At 24- and 48-hour postinjection, whole-body imaging was performed using an IVIS system to evaluate biodistribution and targeting. Fluorescence intensity within the regions of interest (ROIs) was quantified to determine targeting efficiency.

Following imaging, mice were cardiac perfused with cold PBS to remove intravascular cells before tissue collection, ensuring that ex vivo imaging and downstream analyses primarily reflected true tissue infiltration. Both the inflamed (left) and noninflamed (right) quadriceps were excised for ex vivo fluorescence imaging. To further assess cellular-level targeting, muscle tissues were enzymatically dissociated into single-cell suspensions, stained with FITC-anti-F4/80 antibody, and analyzed by flow cytometry. Targeting efficiency was calculated as the proportion of DiD^+^ cells in the inflamed tissue, while engraftment efficiency was defined as the percentage of DiD^+^ cells among total F4/80^+^ macrophages.

### Mechanism exploration

#### Transcriptomic sequencing and analysis

Bulk RNA-seq was conducted to investigate the molecular mechanisms underlying the size-dependent migratory behavior of particle-loaded macrophages. Macrophages were first incubated with PLGA particles of different diameters (0.2, 1, and 3 μm) under conditions that yielded comparable particle/drug loads per cell (MΦ, PLGA0.2@MΦ, PLGA1@MΦ, and PLGA3@MΦ). After loading, cells were washed and further cultured in particle-free medium for 6 to 8 hours to allow nanoparticle efflux, mimicking the circulation phase of adoptively transferred macrophages before tissue recruitment. Total RNA was extracted and subjected to paired-end sequencing (PE150) on the DNBSEQ platform (BGI, ISO/IEC 17025-certified). Each sample yielded approximately 6 Gb of clean data, with ≥85% of bases having a quality score ≥ Q30 and a coverage depth of 30×. Raw sequencing data were processed using SOAPnuke for quality control, aligned to the reference genome using HISAT2, and analyzed for differential gene expression with DESeq2. GO enrichment analysis (significance threshold: *q* ≤ 0.0001) and KEGG pathway enrichment (*q* ≤ 0.01) were performed using the Phyper package (hypergeometric test).

#### Intracellular ROS detection

To evaluate oxidative stress responses associated with different particles, intracellular ROS levels were measured using the DCFH-DA fluorescent probe (10 μM). Macrophages were incubated with the probe for 30 min at 37°C, followed by fluorescence quantification using a microplate reader (excitation/emission: 488/525 nm).

#### Cytoskeletal remodeling

To assess cytoskeletal organization, cells were fixed and stained with Phalloidin-iFluor 647 (Abcam, catalog no. ab176759) to visualize F-actin structures. CLSM was used for imaging, and ImageJ software was used to quantify cell spreading area and fluorescence intensity as indicators of cytoskeletal remodeling.

#### Pathway validation

For gene expression validation, total RNA was extracted using a column-based RNA isolation kit (TransGen Biotech, catalog no. ER111-01) and reverse-transcribed into cDNA. Gene expression was analyzed with SYBR-Green qPCR Master mix using Thermo ABI QuantStudio 6. Cycling conditions were as follows: UDG activation at 50°C for 2 min, initial denaturation at 95°C for 30 s, followed by 40 cycles of denaturation at 95°C for 15 s and annealing/extension at 60°C for 30 s. Gene-specific primers (listed in table S1) were used, with glyceraldehyde-3-phosphate dehydrogenase (GAPDH) serving as the internal control. Relative gene expression levels were calculated using the 2^–ΔΔ^Ct method.

#### Protein expression of CXCR4, ITGB1, P-Akt, and MMP9

Surface expression of CXCR4 and ITGB1 was quantified by flow cytometry. In brief, cells were washed with PBS containing 1% bovine serum albumin and incubated with PE–anti-CXCR4 (eBioscience, catalog no. 12-9991-81, clone 2B11, lot no. 3145064) or APC–anti-ITGB1 (CD29) (eBioscience, catalog no. 17-0291-80, clone eBioHMb1-1, lot no. 3234685) at 4°C for 30 min. After washing, samples were analyzed using a flow cytometer, and data were processed using FlowJo software.

Phosphorylated Akt (P-Akt) and MMP9 protein levels were evaluated by Western blot analysis. Total cellular proteins were extracted with a column-based extraction kit (Seven Biotech, catalog no. SW203), supplemented with protease and phosphatase inhibitors (Epizyme Biotech, catalog no. GRF103). Protein concentration was measured using a BCA assay (ServiceBio, Epizyme Biotech G2026). Equal amounts of protein were separated by gradient-like polyacrylamide gel electrophoresis (Epizyme Biotech, catalog no. PG610) and transferred onto polyvinylidene difluoride membranes. After blocking with a protein-free rapid blocking buffer (Epizyme Biotech, catalog no. PS108), the membranes were incubated overnight at 4°C with primary antibodies targeting P-Akt (Cell Signaling Technology, catalog no. 4060, lot no. 27), MMP9 (ABclonal Technology, catalog no. A0289, lot no. 1151690201), and GAPDH (Servicebio, catalog no. ZB15004-HRP, lot no. AC251027003) as the loading control. Membranes probed with anti–P-Akt and anti-MMP9 antibodies were subsequently incubated with a horseradish peroxidase–conjugated secondary antibody (Epizyme Biotech, catalog no. LF102) after three washes with tris-buffered saline with Tween 20. Protein bands were detected using enhanced chemiluminescence (Guangzhou Biolight Biotechnology, GelView6000proII) and quantified by densitometric analysis with ImageJ software.

#### ROS scavenging with NAC and functional rescue assays

To evaluate the causal contribution of ROS to the migration defects of nanoparticle-loaded macrophages, loss-of-function experiments were performed using the ROS scavenger NAC (Aladdin, catalog no. N103973). Macrophages were assigned to five groups: untreated control (MΦ), PLGA0.2@MΦ, PLGA0.2@MΦ + NAC (1 mM), PLGA0.2@MΦ + NAC (5 mM), and NAC only. For NAC treatment, NAC was added to the culture medium 2 hours before particle incubation and was present throughout 4 hours of particle loading. After the indicated treatments, intracellular ROS levels were quantified as described before. To assess molecular rescue, the surface expression of CXCR4 and ITGB1 was analyzed by flow cytometry with the presence of 1 mM NAC. Functional rescue of migration was evaluated using a transwell assay with the presence of 1 mM NAC.

### Animal model of local inflammation

#### IBD model

To induce acute colitis, male C57BL/6 mice (8 to 10 weeks old) were provided with drinking water containing 3 to 3.5% (w/v) DSS (molecular weight: 36 to 50 kDa; MP Biomedicals, catalog no. MP216011001) ad libitum for seven consecutive days. Disease progression was monitored daily based on body weight loss, stool consistency, and fecal bleeding.

#### In vivo targeting efficiency

IBD mice were randomly divided into three groups (*n* = 4 per group), receiving equal numbers of DiD-labeled macrophages (MΦ), DexP0.2@MΦ, or DexP3@MΦ via tail vein injection. At 24-hour postinjection, mice were cardiac perfused with cold PBS to remove intravascular cells before tissue collection, then colons were excised and subjected to in vivo imaging using an IVIS. Fluorescence intensity in defined ROIs was quantified to evaluate macrophage accumulation. Colon tissues were subsequently homogenized; DiD signal was measured using a fluorescence spectrophotometer, and drug content was determined by LC-MS.

#### Therapeutic efficacy

Mice with IBD were randomly divided into five groups: A: IBD (untreated); B: blank MΦ; C: DexP0.2@MΦ; D: DexP3@MΦ; and E: DexP3 MPs. Healthy mice were used as normal controls. Disease severity was evaluated daily using the DAI, calculated on the basis of the following: body weight loss (0: <1%; 1: 1 to 5%; 2: 5 to 10%; 3: 10 to 15%; and 4: >15%), stool consistency (0: normal; 2: loose stool; and 4: diarrhea), and fecal bleeding (0: none; 2: occult blood; and 4: gross bleeding). At the experimental end point, colons were harvested and measured for length as an indicator of inflammation severity. Tissues were then processed into single-cell suspensions for macrophage polarization analysis by flow cytometry using FITC–anti-F4/80, PE–anti-CD206, and PE/Cyanine7–anti-CD80 (BioLegend, catalog no. 104733, clone 16-10A1, lot no. B413526). Parallel colon segments were sectioned for histopathological assessment by hematoxylin and eosin (H&E) and Alcian Blue staining to evaluate inflammatory infiltration and epithelial damage. The remaining colon tissues were homogenized, and the total protein concentration of each homogenate was determined using a BCA protein assay before ELISA. Cytokine levels were subsequently quantified by ELISA and normalized to the total protein content of the corresponding tissue homogenate.

#### Safety evaluation

Healthy female BALB/c mice (6 to 8 weeks) were randomly divided into four groups (*n* = 3) and intravenously injected with PBS, blank MΦ, DexP0.2@MΦ, and DexP3@MΦ (2 × 10^6^ cells in 100 μl of PBS). After 24 hours, the blood samples were collected for a complete blood count test and biochemistry analysis, including ALT and AST, BUN, and Cr for indication of liver and kidney function, respectively (fig. S23). Major organs (heart, liver, spleen, lung, and kidney) were collected and fixed with 4% PFA for H&E staining (fig. S24).

### Statistics and reproducibility

All experiments were independently repeated at least twice. All data points represent biological replicates and are presented as mean ± SD. Statistical analyses were performed using Origin (version 10.15, 2024). Sample sizes were determined on the basis of prior experience, preliminary experiments, and conventions in the field. One-way analysis of variance (ANOVA) with Tukey’s multiple comparisons test, Brown-Forsythe and Welch ANOVA tests followed by Dunnett’s T3 multiple comparisons, or two-tailed unpaired Student’s *t* tests were applied, as appropriate. Statistical significance was defined as *P < 0.05, **P < 0.01, ***P < 0.001, and ****P < 0.0001. Experimental reproducibility is indicated in the figure legends. No data were excluded from the analyses.

### Schematic illustrations

Schematic illustrations were created with Biorender.com.
