# Supplementary material for: Loading is not delivering: Intracellular drug retention and migration capacity govern macrophage-based drug delivery
Source: Sci Adv. 2026 Jul 24;12(30):eaec0429. doi: 10.1126/sciadv.aec0429 (PMC13398538; doi:10.1126/sciadv.aec0429)
Supplement: Supplementary file 1 — Figs. S1 to S24 Table S1 [file sciadv.aec0429_sm.pdf]

Science Advances
AAAS

Supplementary Materials for

Loading is not delivering: Intracellular drug retention and migration capacity govern macrophage-based drug delivery

Yao Huang *et al.*

Corresponding author: Wei Li, liwei2023@hust.edu.cn, wei.li@helsinki.fi

Sci. Adv. **12**, eaec0429 (2026)
DOI: 10.1126/sciadv.aec0429

This PDF file includes:

Figs. S1 to S24
Table S1

Supplementary Figures

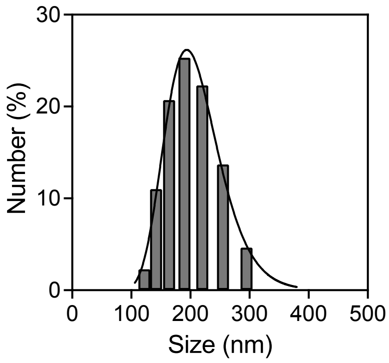

Fig. S1 Size distribution of PLGA nanoparticles determined by dynamic light scattering.

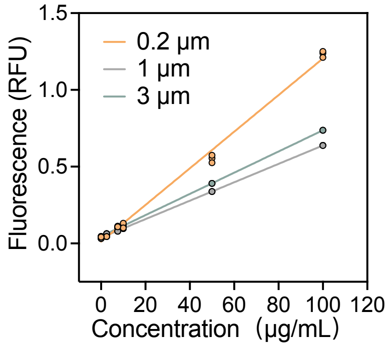

Fig. S2 Standard curves of Rhodamine B-labeled PLGA particles (n = 3):

0.2 μm PLGA-RhB: $y = 0.012x + 0.01$, $R^2 = 0.995$,

1 μm PLGA-RhB: $y = 0.006x + 0.03$, $R^2 = 0.999$,

3 μm PLGA-RhB: $y = 0.007x + 0.05$, $R^2 = 1.000$.

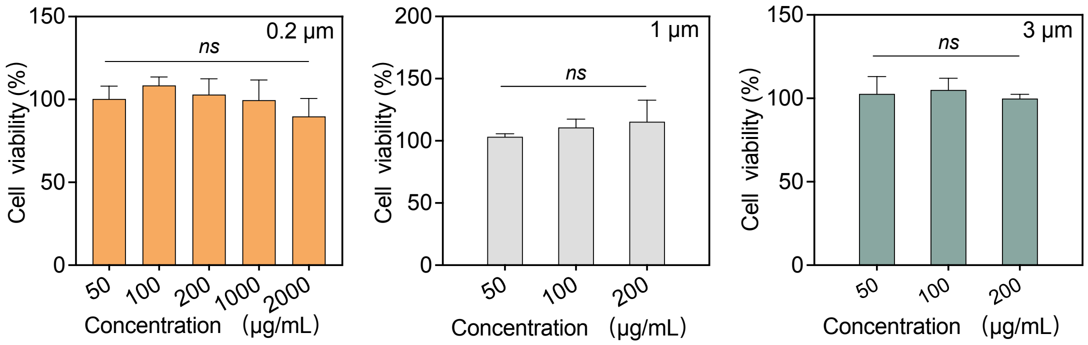

Fig. S3 Viability of PLGA particle-loaded macrophages compared to untreated macrophages (n = 5).

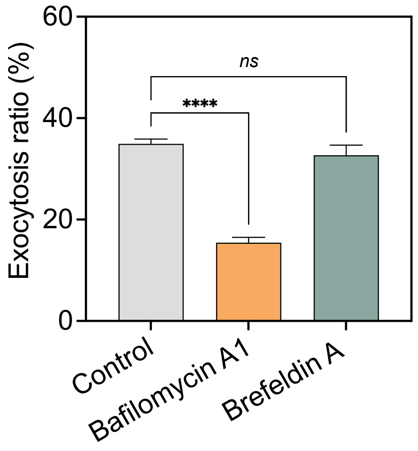

Fig. S4 Effects of Bafilomycin A1 (lysosome-mediated exocytosis inhibitor) and Brefeldin A (ER–Golgi secretory pathway inhibitor) on nanoparticle exocytosis ($n = 3$). ****$P<0.0001$; data are shown as mean ± SD, statistical significance was analyzed using a one-way ANOVA with Tukey's multiple comparisons.

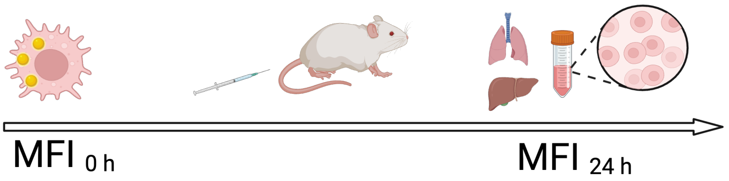

Fig. S5 Schematic illustration of the *in vivo* particle exocytosis experiments. Created in BioRender. Huang, Y. (2026) https://BioRender.com/ob3qa1e.

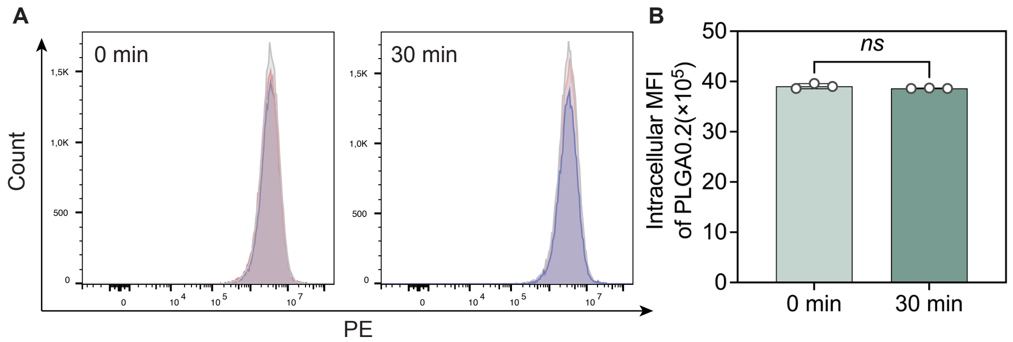

Fig. S6 Intracellular particles remain stable over 30 min interval between cell collection and adoptive transfer. **(A)** Representative flow cytometry histograms showing the intracellular signal of PLGA-RhB nanoparticles at 0 min and 30 min. **(B)** Quantification of intracellular PLGA means fluorescence intensity (MFI, PE-A) at 0 min and 30 min

(n = 3). Data are presented as mean ± SD, and statistical significance was analyzed using a two-tailed unpaired Student's t test.

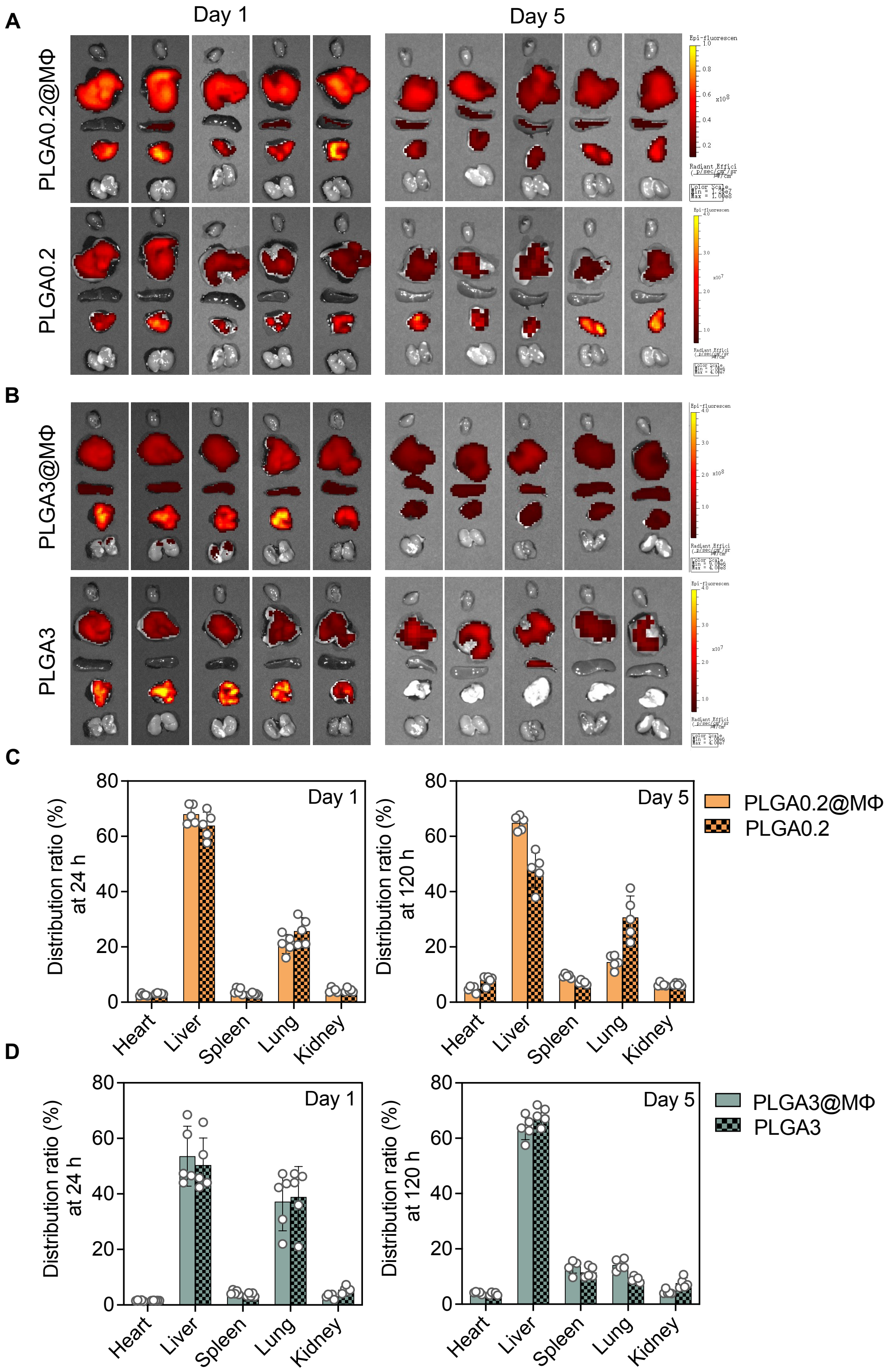

Fig. S7 *In vivo* biodistribution of PLGA0.2@MΦ and PLGA3@MΦ. (**A**) Representative IVIS images showing the *in vivo* distribution of DiD-labeled

PLGA0.2@MΦ macrophages and rhodamine B–labeled PLGA0.2 nanoparticle at day 1 and day 5 post-adoptive transfer. (**B**) Representative IVIS images showing the *in vivo* distribution of DiD-labeled PLGA3@MΦ macrophages and rhodamine B–labeled PLGA3 microparticles at day 1 and day 5 post-adoptive transfer. (**C**) Quantitative analysis of the tissue distribution of PLGA0.2@MΦ macrophages and PLGA0.2 nanoparticle cargo over time at (A) (*n* = 5). (D) Quantitative analysis of the tissue distribution of PLGA3@MΦ macrophages and PLGA3 microparticles over time at (B) (*n* = 5). Organs in (A-B) were arranged from top to bottom as heart, liver, spleen, lung, and kidney.

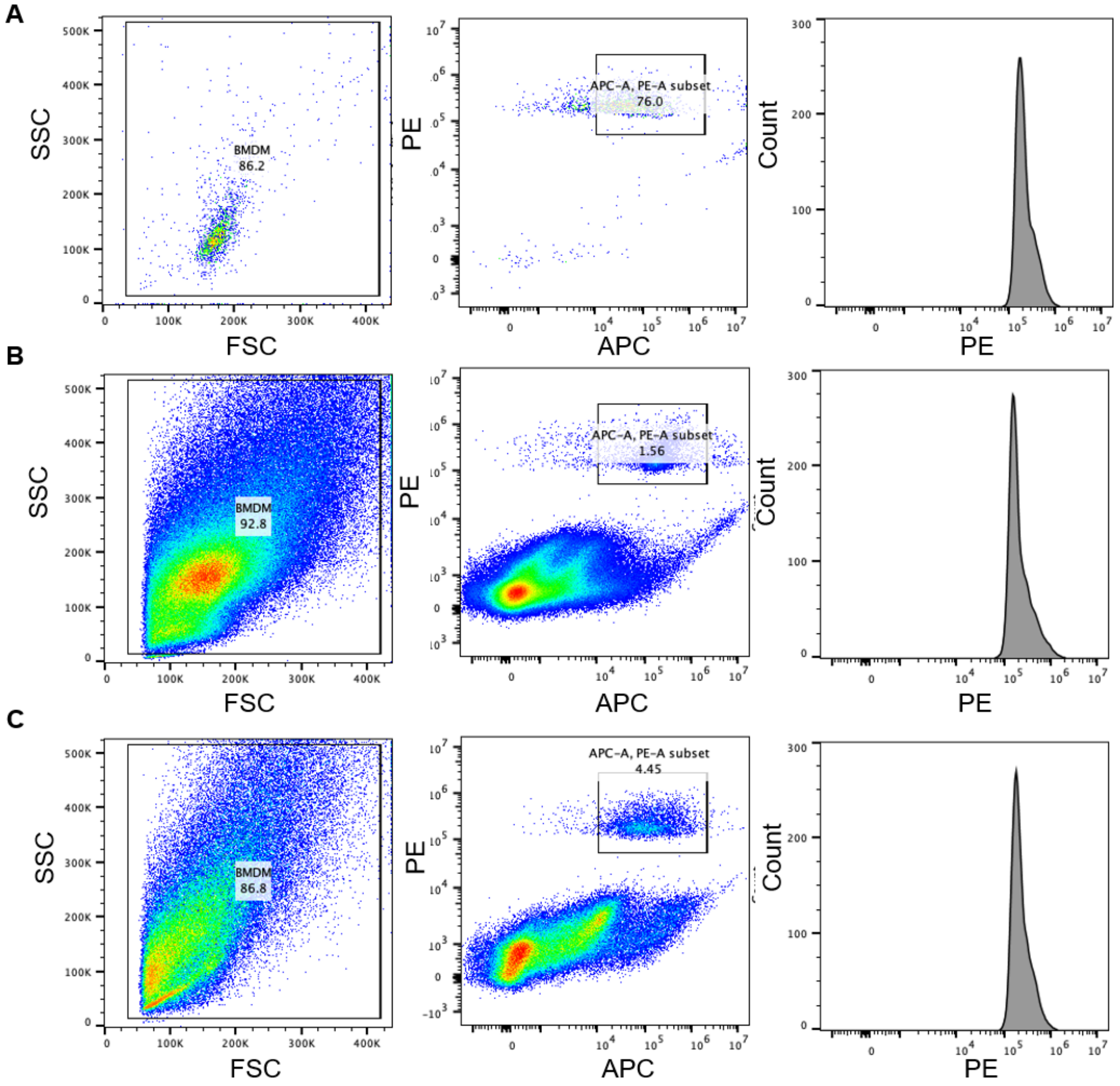

Fig. S8 Flow cytometry plots showing DiD-labelled BMDMs loaded with 3 μm PLGA microparticles (PLGA3@MΦ) before and after adoptive transfer. (**A**) PLGA3@MΦ

prior to adoptive transfer (0 h). (**B, C**) PLGA3@MΦ isolated from the liver (B) and lung (C) 24 h after systemic administration. DiD fluorescence (APC channel) identifies adoptive macrophages, while PLGA particles are detected via their rhodamine B fluorescence (PE channel). A reduction in the mean fluorescence intensity (MFI) of the PE signal in DiD⁺ macrophages reflects particle exocytosis *in vivo*.

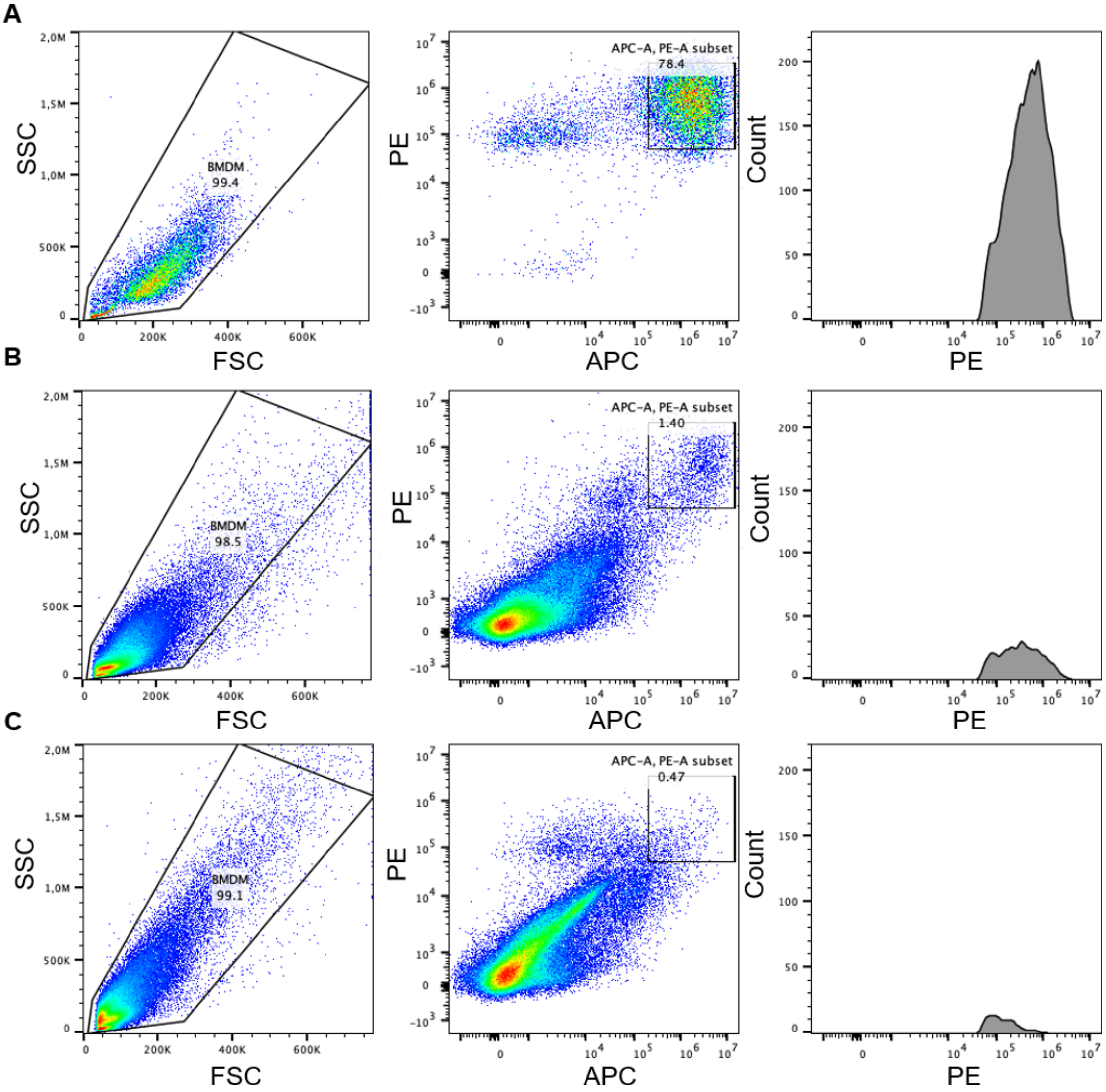

Fig. S9 Flow cytometry plots showing DiD-labelled BMDMs loaded with 0.2 µm PLGA nanoparticles (PLGA0.2@MΦ) before and after adoptive transfer. (**A**) PLGA0.2@MΦ prior to adoptive transfer (0 h). (**B, C**) PLGA0.2@MΦ isolated from the liver (B) and lung (C) 24 h after systemic administration. DiD fluorescence (APC channel) identifies adoptive macrophages, while PLGA particles are detected via their rhodamine B fluorescence (PE channel). A reduction in the MFI of the PE signal in

DiD$^+$ macrophages reflect particle exocytosis *in vivo*.

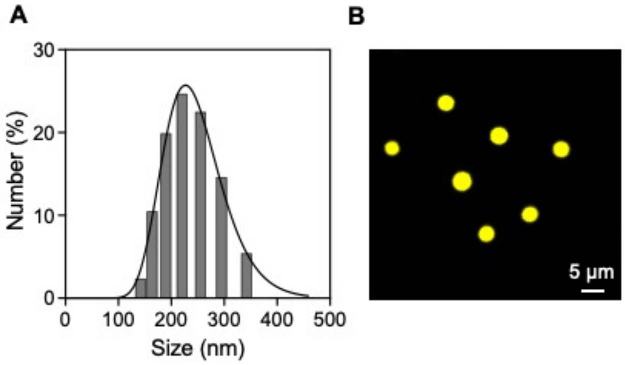

Fig. S10 Characterization of DexP particles. (**A**) Size distribution of 0.2 μm DexP nanoparticles determined by dynamic light scattering. (**B**) CLSM image of 3 μm DiI-labelled DexP microparticles (scale bar: 5 μm).

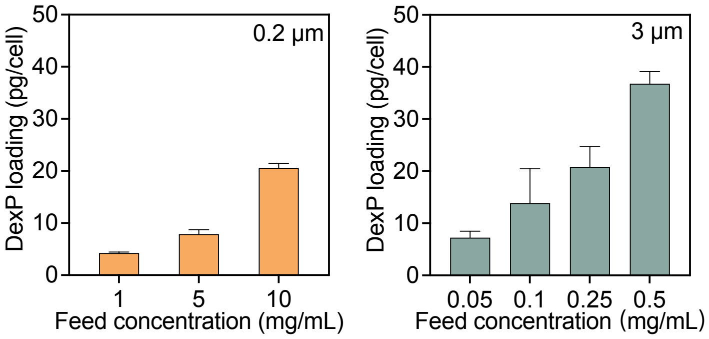

Fig. S11 Intracellular drug loading of 0.2 μm nanoparticles and 3 μm microparticles of DexP (n = 3). Feed doses of 10 mg/mL for the 0.2 μm nanoparticles and 0.25 mg/mL for the 3 μm microparticles were used in subsequent experiments.

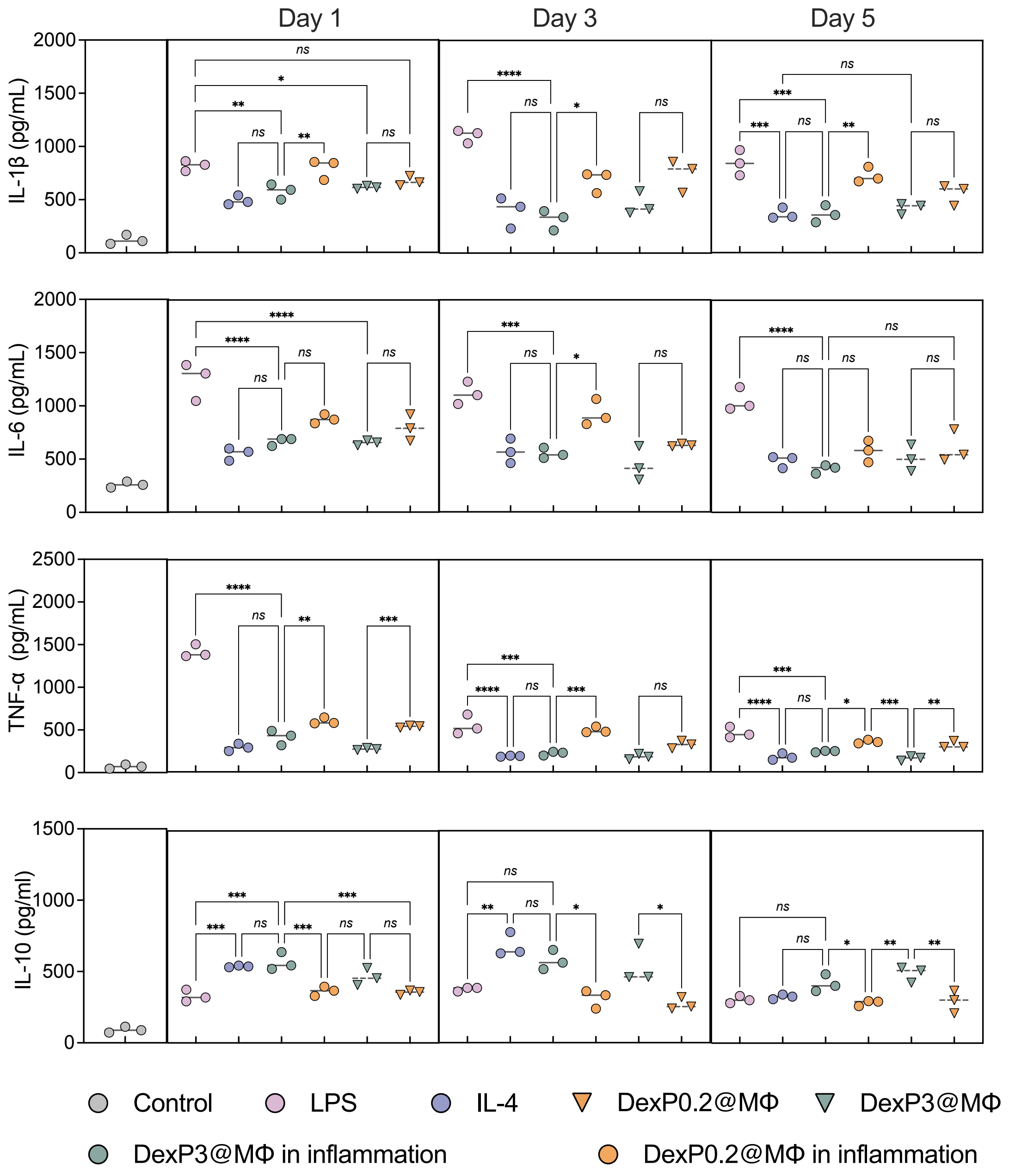

Fig. S12 Cytokine concentrations in the cell culture medium following macrophage polarization ($n = 3$). *P<0.05, **P <0.01, ***P <0.001, ****P<0.0001; data are shown as mean ± SD; statistical significance was analyzed using a one-way ANOVA with Tukey's multiple-comparison test.

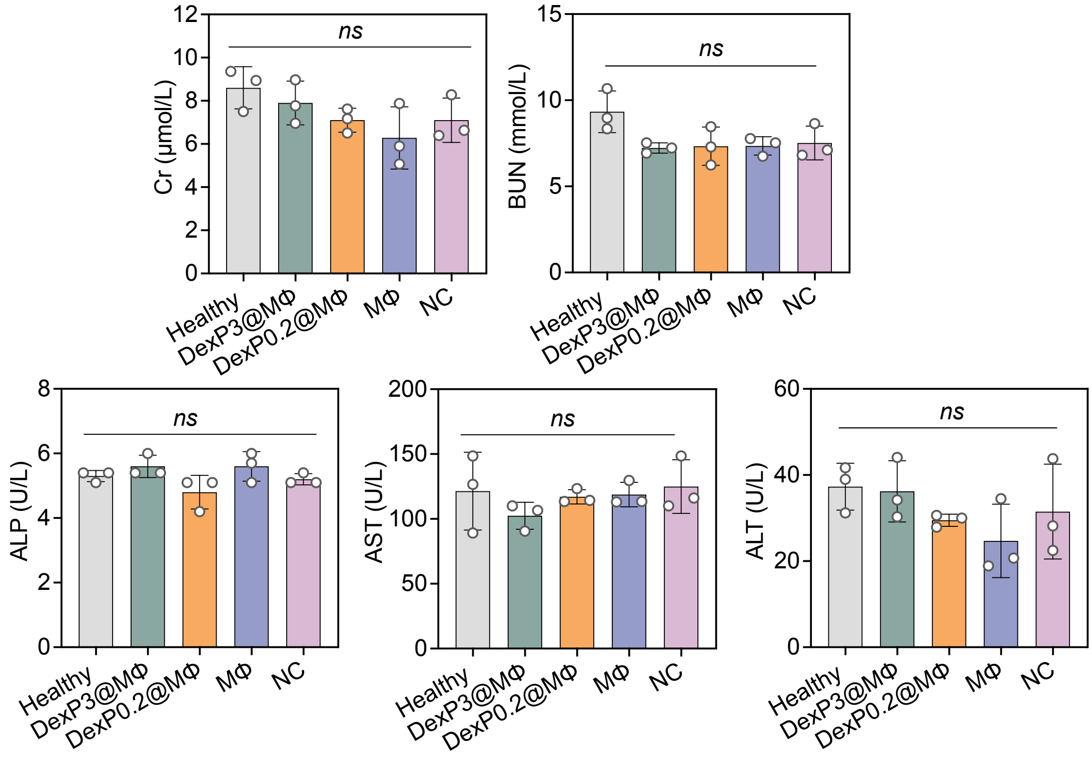

Fig. S13 Serum markers of kidney (BUN, Cr) and liver (ALT, AST, ALP) function (*n* = 3). Data are shown as mean ± SD; statistical significance was analyzed using a one-way ANOVA with Tukey's multiple comparisons.

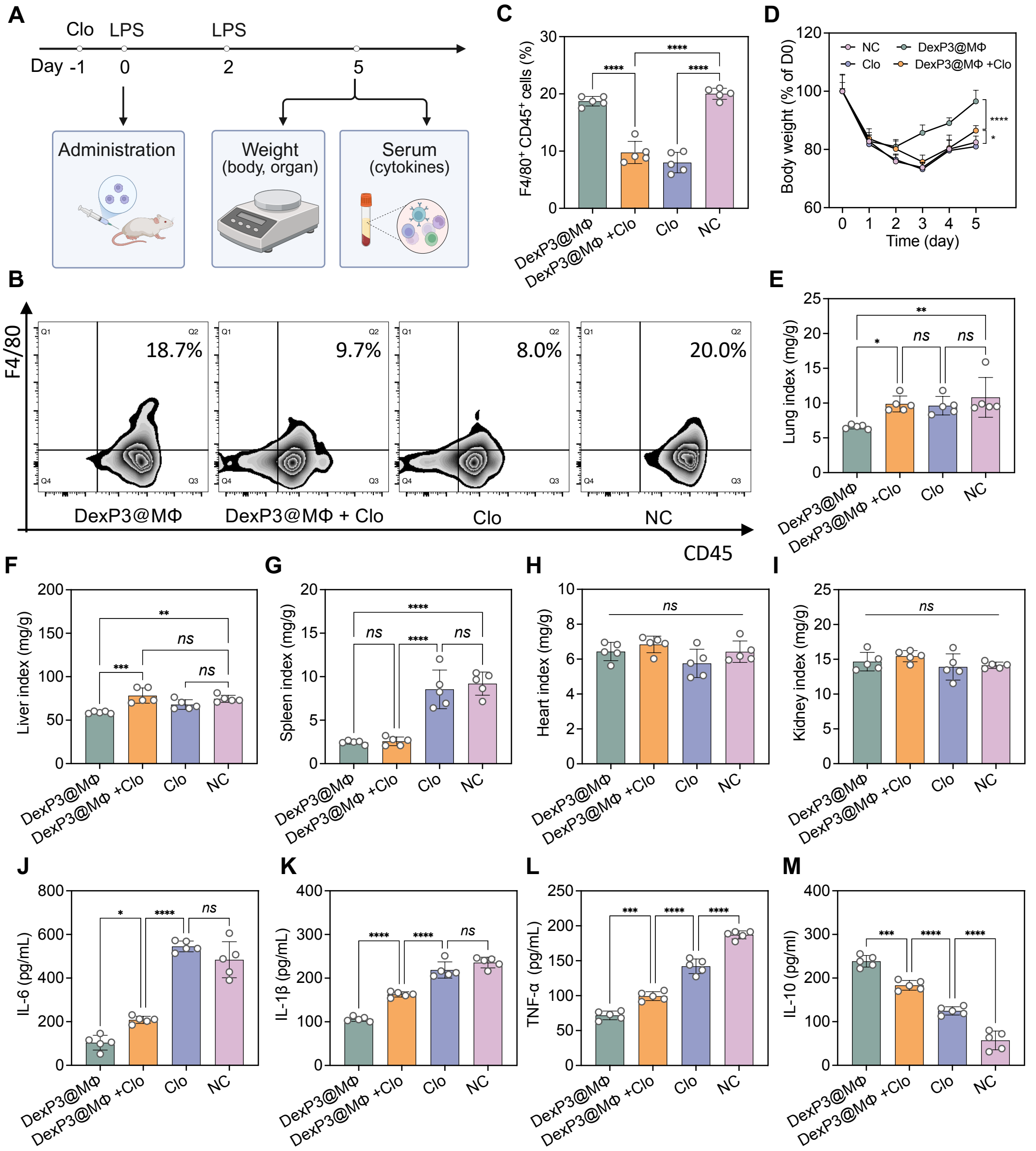

Fig. S14 Macrophage depletion reveals combined contributions of adoptively transferred and endogenous macrophages to therapeutic efficacy in systemic inflammation. Data shown in Fig. S14 and Fig. 3 were obtained from independent experiments and should be interpreted within their respective experimental contexts. (**A**) Schematic illustration of the macrophage depletion experiment in the systemic inflammation model. Created in BioRender. Huang, Y. (2026) https://BioRender.com/srau444. (**B**) Representative flow cytometry zebra plots showing splenic macrophage populations across experimental groups. (**C**) Quantification of splenic macrophage frequencies determined by flow cytometry (*n* = 5). (**D**) Body weight of mice after specified treatment (*n* = 5). (**E–I**) Organ index of major organs (*n* = 5). (**J–M**) Serum levels of pro-inflammatory (IL-6, IL-1β, TNF-α)

and anti-inflammatory (IL-10) cytokines (n = 5). *P<0.05, **P<0.01, ***P<0.001, ****P<0.0001; Data are shown as mean ± SD, statistical significance was analyzed using a one-way ANOVA with Tukey's multiple comparisons (C–M).

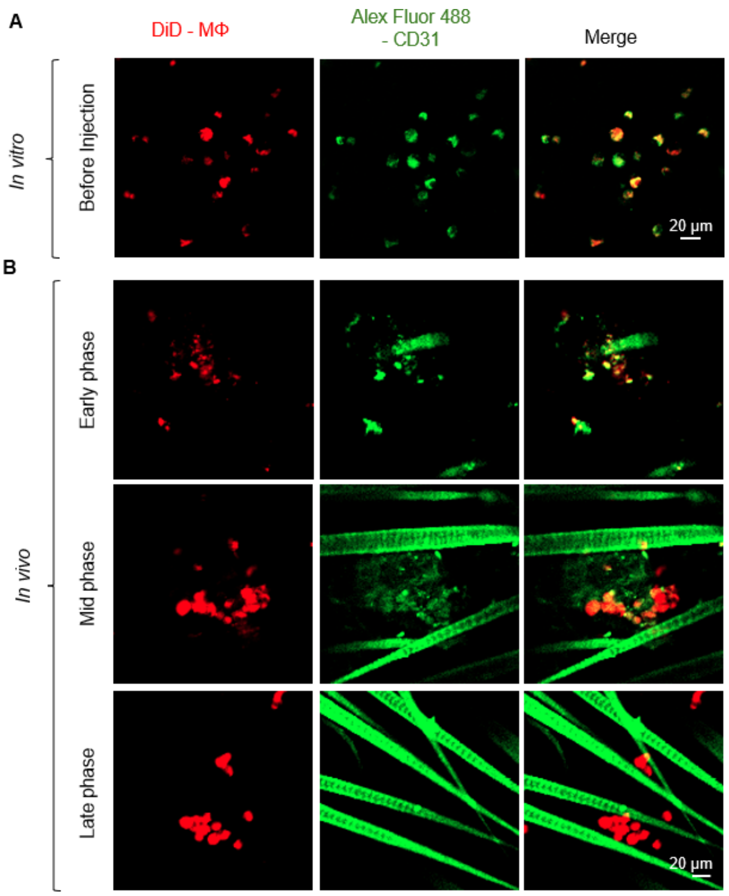

Fig. S15 Macrophage-associated anti-CD31 fluorescence is transient *in vivo* (scale bar: 20 μm). BMDMs were pre-incubated *in vitro* with Alexa Fluor 488-anti-CD31 antibody, followed by extensive washing to remove unbound antibody. (**A**) CLSM confirmed the presence of cell-associated green fluorescence on DiD-labeled macrophages before injection. The pre-labeled macrophages were then subcutaneously injected into the ears of mice without systemic CD31 antibody administration. (B) *In vivo* CLSM showed that macrophage-associated green fluorescence was detectable at early time points but decreased over time. These data indicate that anti-CD31-associated fluorescence on macrophages is transient *in vivo* and may help explain the transient green signal observed near macrophages during intravital imaging in Fig. 4G, H.

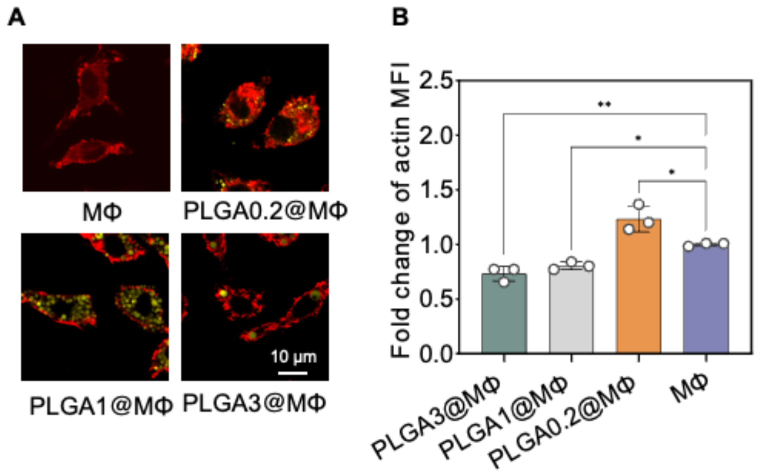

Fig. S16 Image of F-actin (phalloidin staining) by CLSM (**A**) and quantified F-actin MFI in macrophages (**B**) (n = 3; scale bar: 10 μm). **P*<0.05, ***P*<0.01; the data are shown as mean ± SD, statistical significance was analyzed using a one-way ANOVA with Tukey's multiple comparisons

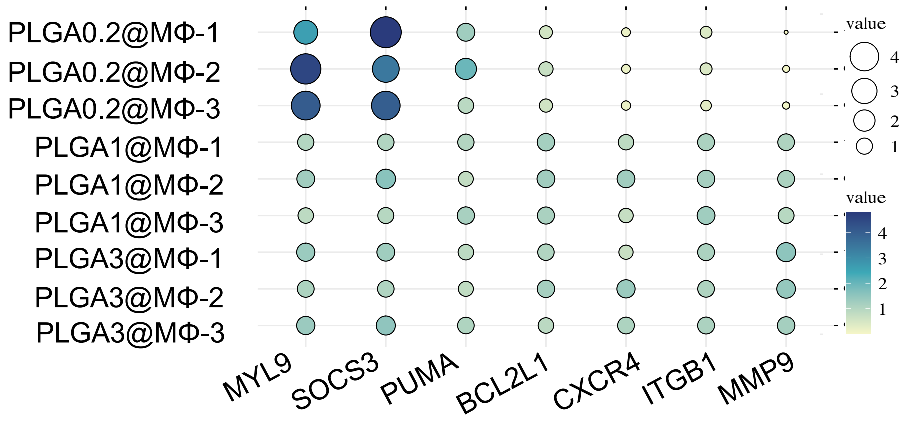

Fig. S17 Bubble plot of qPCR-validated genes. Bubble size and color intensity reflect fold-changes compared to untreated macrophages.

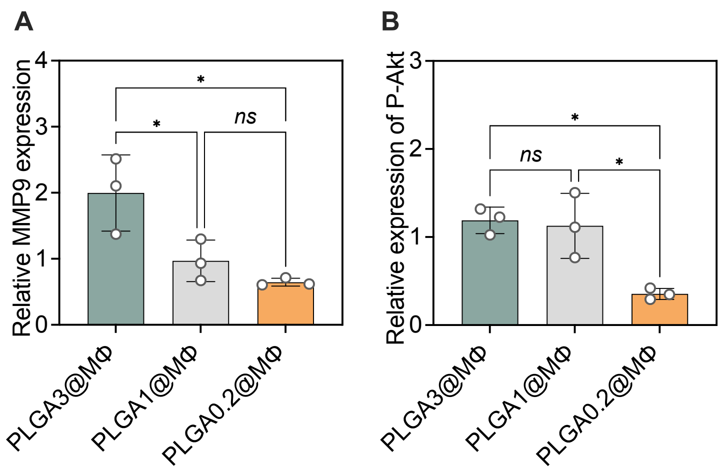

Fig. S18 Quantification of Western blot analysis showing relative expression levels of MMP9 and phosphorylated Akt (P-Akt). Protein intensities were normalized to GAPDH from three independent experiments (*n* = 3). **P*<0.05; the data are shown as mean ± SD, statistical significance was analyzed using a one-way ANOVA with Tukey's multiple comparisons.

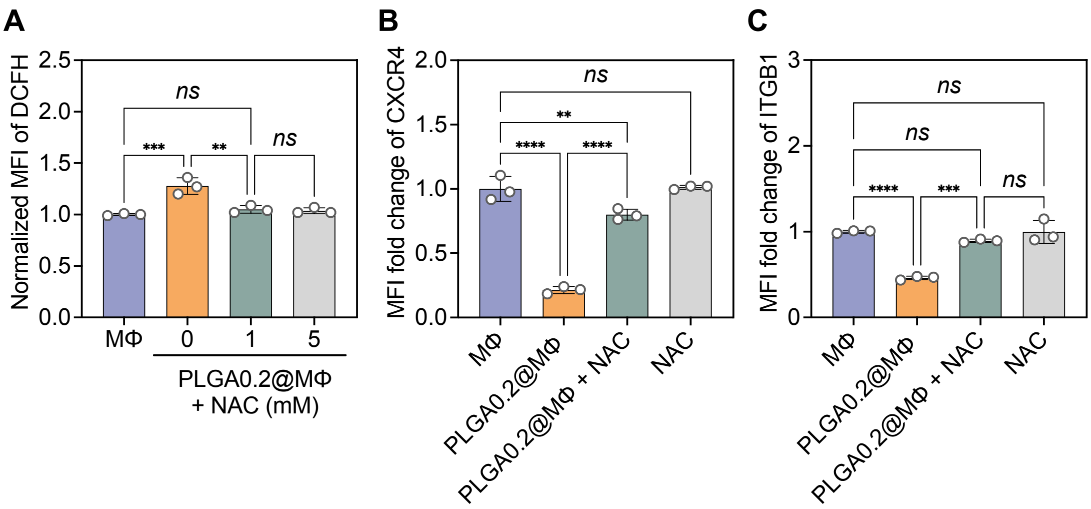

Fig. S19 NAC reverses ROS-mediated migration defects in PLGA0.2-loaded macrophages. For direct comparison, the MΦ and PLGA0.2@MΦ groups from Fig. 5F, G are included in Fig. S19 B, C. (**A**) Quantification of intracellular ROS levels (*n* = 3). (**B–C**) Surface expression of CXCR4 (B) and ITGB1 (C) measured by flow cytometry (*n* = 3). ***P*<0.01, *** *P*<0.001, *****P*<0.0001; the data are shown as mean ± SD; statistical significance was analyzed using a one-way ANOVA with Tukey's multiple comparisons.

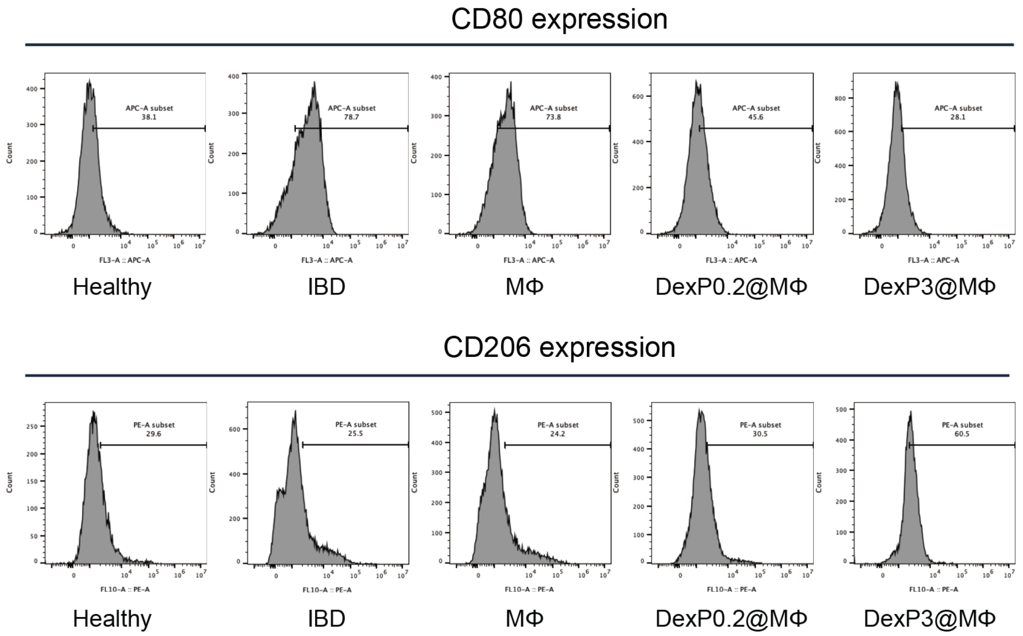

Fig. S20 Flow cytometry histograms of CD80 and CD206 expression in colonic macrophages isolated from mice under different treatment conditions. Macrophages were gated as F4/80$^+$ cells.

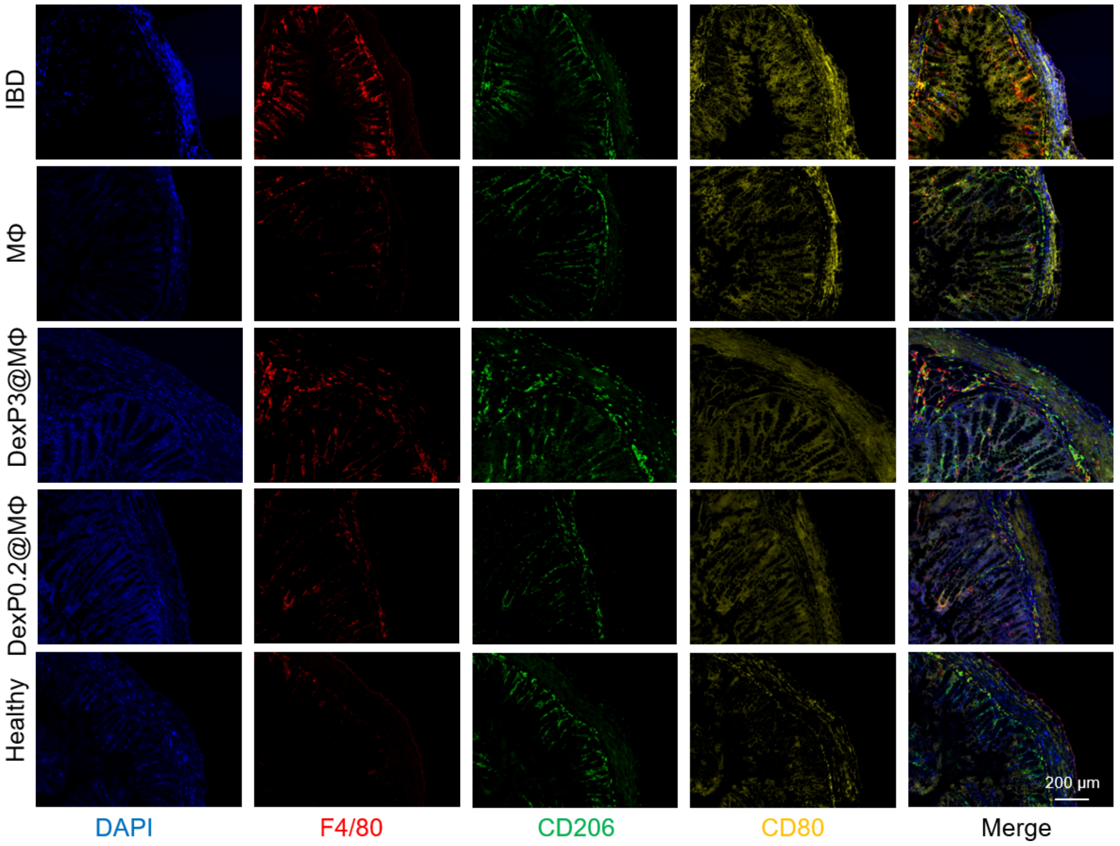

Fig. S21 Immunofluorescence staining of colon sections (scale bar: 200 μm). Nuclei

were stained with DAPI (blue), macrophages with F4/80 (red), M2 marker CD206 (green), and M1 marker CD80 (yellow).

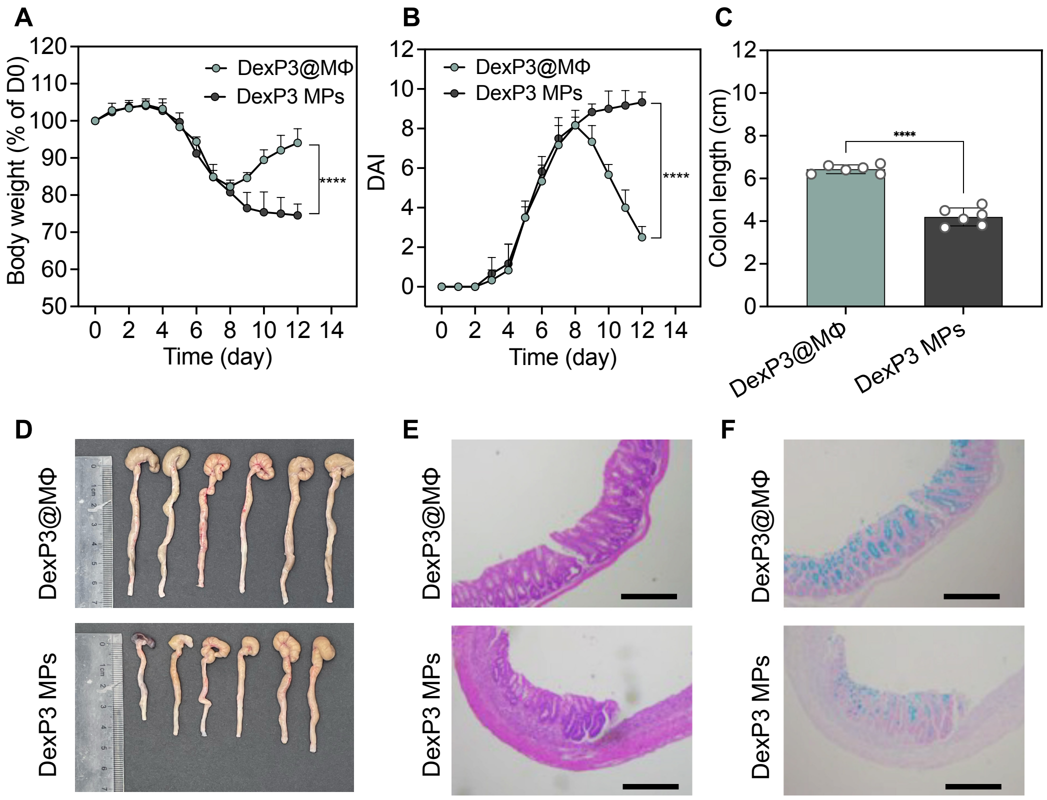

Fig. S22 Macrophage-mediated delivery is critical for therapeutic efficacy in DSS-induced IBD. Data were obtained from an independent experiment performed to compare DexP3@MΦ with free DexP3 microparticles and should be interpreted within this experimental cohort. (**A**) Body weight changes over the treatment course (*n* = 6). (**B**) Disease activity index (DAI) scoring over time (*n* = 6). (**C, D**) Colon length (C) and colon images (D). (**E, F**) Histopathological analysis: (E) H&E staining (inflammatory infiltrate scoring) and (F) Alcian blue (AB) staining for goblet cell preservation (scale bars: 500 μm). *****P*<0.0001; the data are shown as mean ± SD, statistical significance was analyzed using a two-tailed unpaired Student's *t* test.

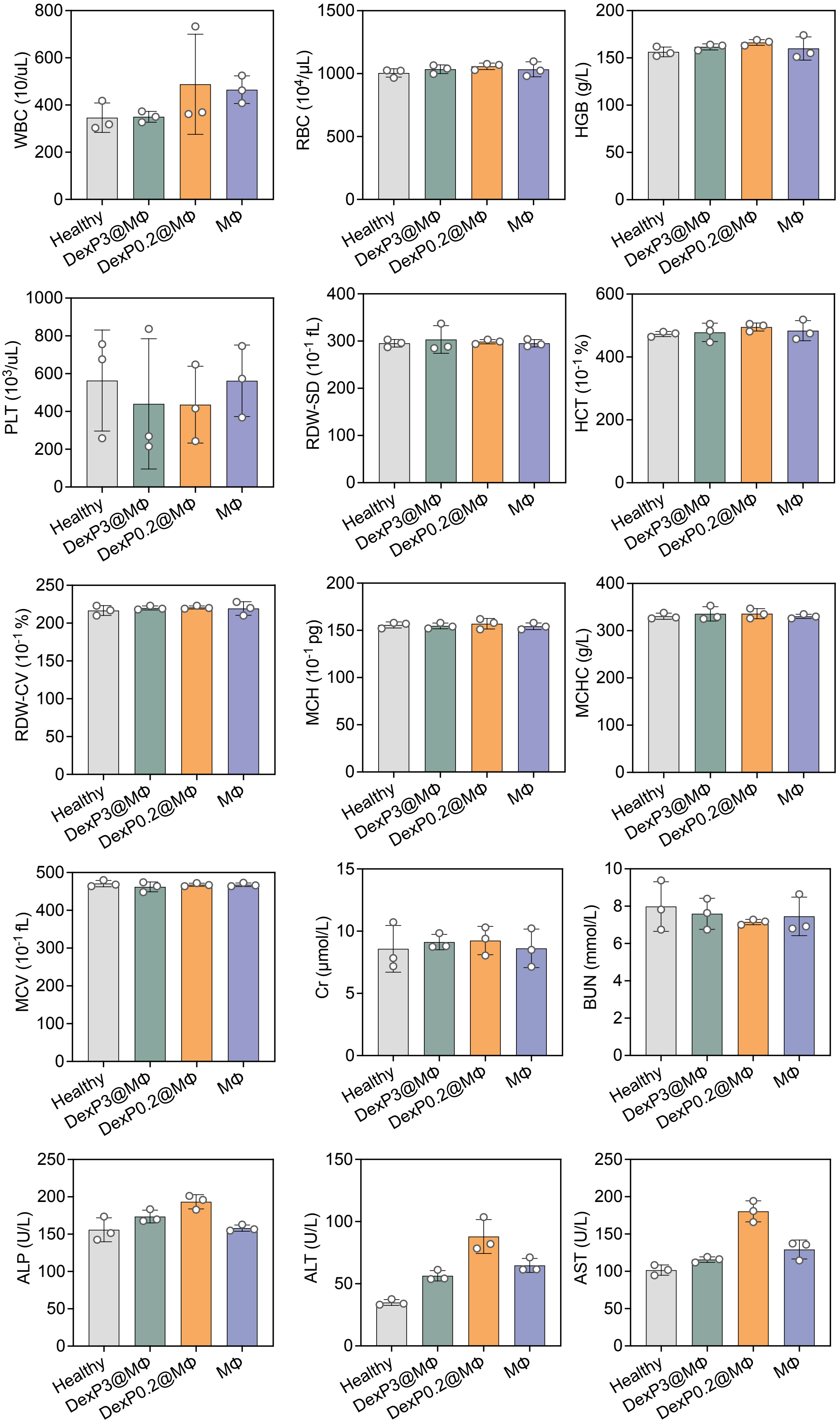

WBC (10/uL)
RBC (10^4/μL)
HGB (g/L)
PLT (10^3/uL)
RDW-SD (10^-1 fL)
HCT (10^-1 %)
RDW-CV (10^-1 %)
MCH (10^-1 pg)
MCHC (g/L)
MCV (10^-1 fL)
Cr (μmol/L)
BUN (mmol/L)
ALP (U/L)
ALT (U/L)
AST (U/L)
Healthy
DexP3@MΦ
DexP0.2@MΦ
MΦ

Fig. S23 Safety evaluation of treatment. Hematological parameters and serum biochemical markers of liver and kidney function were assessed 24 hours post-administration (*n* = 3). No overt hematological or biochemical abnormalities were observed compared to controls, indicating minimal hematological and organ toxicity.

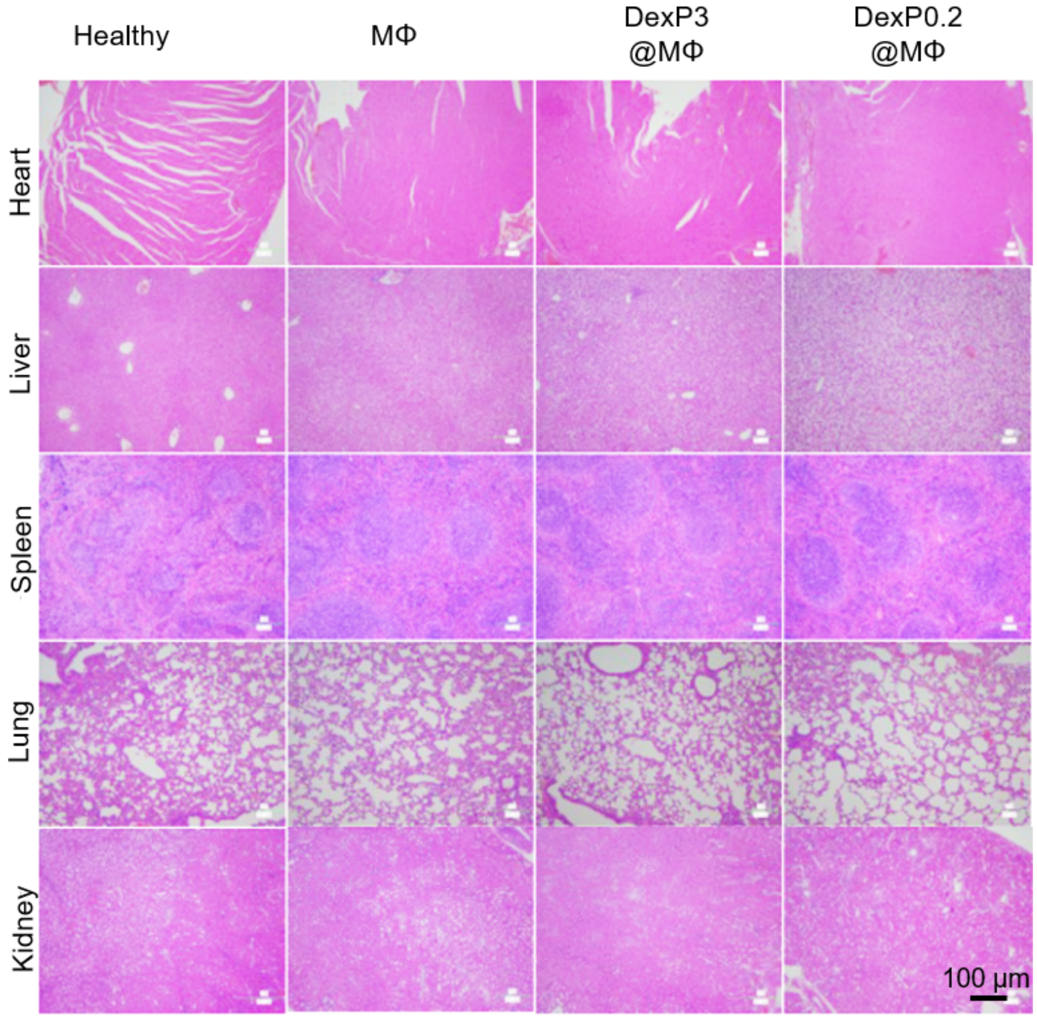

Fig. S24 Histological safety evaluation post-treatment. Representative H&E-stained sections of heart, liver, spleen, lung, and kidney harvested 24 hours after treatment (scale bar: 100 μm). No notable histological abnormalities, such as inflammation, necrosis, or structural damage, were observed compared to healthy controls, indicating good biocompatibility and systemic safety.

Supplementary Table

Table S1. Primer sequences for PCR amplification

Primer names	Sequences (5'-3')
M-Myl9-S	AGGCCAAGACCACCAAGAAG
M-Myl9-A	TGCCCTCCAGATACTCGTCT
M-Socs3-S	GGTCACCCACAGCAAGTTTCC
M-Socs3-A	GTCACGGCGCTCCAGTAGAAT
M-puma-S	AGCGGCGGAGACAAGAAGA
M-puma-A	GGAGGAGTCCCATGAAGAGATTG
M-BCL2L1-S	GGCTTTGTTTCCCTTGCCTC
M-BCL2L1-A	CTCCTGTCTTGCGGTTCTGT
M-CXCR4-S	CCTTTACCCCGATAGCCTGTG
M-CXCR4-A	ATCCTTGCTTGATGACCCCCA
M-ITGB1-S	AATTTGATTCCTAAGTCAGCAGTGG
M-ITGB1-A	CTGAAGAAAGAGAGTTGTAGGCATC
M-MMP9-S	GCTGGCAGAGGCATACTTGTAC
M-MMP9-A	GGTGTTCGAATGGCCTTTAGTG
M-GAPDH-S	CCTCGTCCCGTAGACAAAATG
M-GAPDH-A	TGAGGTCAATGAAGGGGTCGT
